# ISB 2001 trispecific T cell engager shows strong tumor cytotoxicity and overcomes immune escape mechanisms of multiple myeloma cells

**DOI:** 10.1038/s43018-024-00821-1

**Published:** 2024-09-11

**Authors:** Laura Carretero-Iglesia, Olivia J. Hall, Jérémy Berret, Daniela Pais, Carole Estoppey, Myriam Chimen, Thierry Monney, Jeremy Loyau, Cyrille Dreyfus, Julie Macoin, Cynthia Perez, Vinu Menon, Isabelle Gruber, Amélie Laurendon, Lydia N. Caro, Girish S. Gudi, Tomomi Matsuura, Piet H. van der Graaf, Stanislas Blein, M. Lamine Mbow, Rebecca Croasdale-Wood, Ankita Srivastava, Michael R. Dyson, Thomas Matthes, Zeynep Kaya, Claire M. Edwards, James R. Edwards, Sophie Maiga, Catherine Pellat-Deceunynck, Cyrille Touzeau, Philippe Moreau, Cyril Konto, Adam Drake, Eugene A. Zhukovsky, Mario Perro, Maria Pihlgren

**Affiliations:** 1 Ichnos Glenmark Innovation, New York, NY USA; 2 Certara UK Limited, Canterbury Innovation Centre, University Road, Canterbury, United Kingdom; 3 Hematology Service, Department of Oncology and Clinical Pathology Service, Department of Diagnostics, University Hospital Geneva, Geneva, Switzerland; 4 Nuffield Department of Orthopaedics, Rheumatology and Musculoskeletal Sciences, Botnar Institute, University of Oxford, Oxford, United Kingdom; 5 Nantes Université, Inserm, CNRS, Université d’Angers, Nantes, France; 6 SIRIC ILIAD, Angers, Nantes, France; 7 Service d’Hématologie Clinique, Unité d’Investigation Clinique, CHU, Nantes, France

**Keywords:** Tumour immunology, Myeloma, Antibody therapy, Cancer immunotherapy, Cancer

## Abstract

Despite recent advances in immunotherapies targeting single tumor-associated antigens, patients with multiple myeloma eventually relapse. ISB 2001 is a CD3^+^ T cell engager (TCE) co-targeting BCMA and CD38 designed to improve cytotoxicity against multiple myeloma. Targeting of two tumor-associated antigens by a single TCE resulted in superior cytotoxic potency across a variable range of BCMA and CD38 tumor expression profiles mimicking natural tumor heterogeneity, improved resistance to competing soluble factors and exhibited superior cytotoxic potency on patient-derived samples and in mouse models. Despite the broad expression of CD38 across human tissues, ISB 2001 demonstrated a reduced T cell activation profile in the absence of tumor cells when compared to TCEs targeting CD38 only. To determine an optimal first-in-human dose for the ongoing clinical trial (NCT05862012), we developed an innovative quantitative systems pharmacology model leveraging preclinical data, using a minimum pharmacologically active dose approach, therefore reducing patient exposure to subefficacious doses of therapies.

## Abstract (web summary)

Perro and colleagues develop a CD3^+^ T cell engager co-targeting BCMA and CD38 to improve immunotherapy for multiple myeloma, demonstrate cytotoxicity in patient-derived samples and murine models and develop a quantitative systems pharmacology model.

## Main

Multiple myeloma (MM) is the second most common hematological malignancy with an estimated worldwide incidence of 160,000 cases in 2020 (ref. ^1^). New therapies, including anti-CD38 monoclonal antibodies and bispecific TCE antibodies have substantially extended patients’ lifespans. Teclistamab^2^, a TCE-targeting B cell maturation antigen (BCMA) on MM cells and CD3ε on T cells, has demonstrated high overall response rates^3–5^. Additional BCMA-targeting TCEs have been explored: EM801 (ref. ^6^) and alnuctamab^7^ utilize a bivalent binding to BCMA, allowing target cell avidity with the anti-CD3ε domain located proximal to the silent Fc^6^. However, patients with relapsed/refractory (r/r) MM continue to progress. Multiple emerging resistance mechanisms, including tumor-associated antigen (TAA) downregulation, account for this.

Simultaneous targeting of two TAAs is a promising approach to prevent escape of tumor cells^8,9^. We explored the co-targeting of BCMA and CD38 on MM cells within a single TCE. ISB 2001 is a trispecific antibody based on the BEAT (Bispecific Engagement by Antibodies based on the TCR) platform^10,11^.

Preclinical toxicology studies require cross-reactivity at similar affinity of all binding domains with animal species antigens. This generally limits first-in-human (FIH) dose selection to the most conservative minimum anticipated biological effect level (MABEL) approach due to lack of relevant animal data. It is therefore important to establish new approaches to increase the starting dose and minimize the number of patients exposed to inactive doses, a guiding principle for dose-escalation phase I trials as per European Medicines Agency and US Food and Drug Administration (FDA) guidelines^12–14^.

Here we show that dual targeting of BCMA and CD38 increases the binding avidity to MM cells and leads to their enhanced killing. This approach also limited the inhibitory impact on tumor killing by soluble factors (soluble BCMA (sBCMA) and APRIL), found at a high level in patients with MM. The architecture and location of the Fab domains of ISB 2001 were optimized to induce strong tumor killing, while minimizing on-target, off-tumor T cell activation and cytokine secretion. In vivo, ISB 2001 was able to induce complete tumor regression in humanized mouse models. ISB 2001 showed superior cytotoxicity of patient-derived r/r MM cells when compared to teclistamab. Finally, we created a quantitative systems pharmacology (QSP) model supporting a minimal pharmacologically active dose (MPAD) approach for the FIH dose calculation, substantially increasing starting dose over MABEL. This innovative approach was accepted by the US FDA and Australian Human Research Ethics Committee, paving the way for similar determination of the FIH dose for other TCEs in preclinical development.

## Results

### Generation of ISB 2001 a CD3, BCMA, CD38-specific antibody

**Fig. 1:**
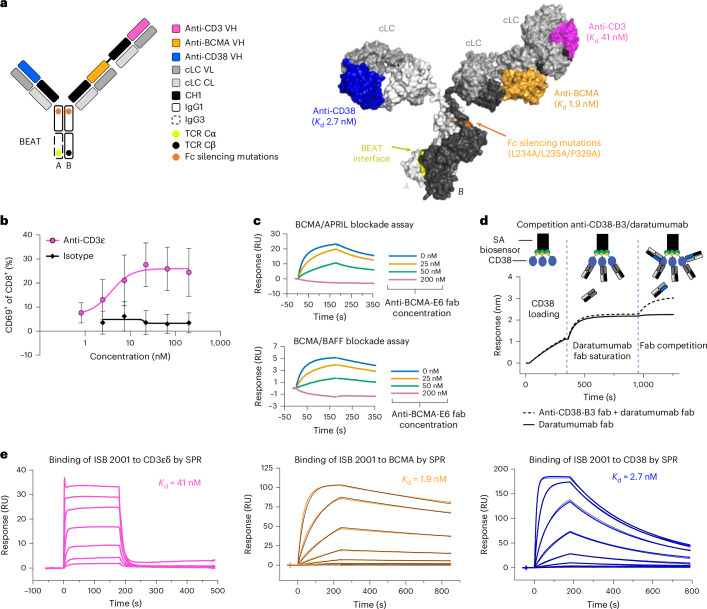
Generation of ISB 2001, a CD3 × BCMA× CD38 trispecific antibody based on the BEAT platform. **a**, Cartoon and structural model of ISB 2001 BEAT. On the cartoon, immunoglobulin domains are shown as rectangles. VH domains of the anti-CD38, anti-BCMA and anti-CD3ε binders are depicted in blue, orange and magenta, respectively. All binders make use of a cLC depicted in gray. Fc-silencing mutations are depicted by the orange dots. The BEAT interface shown in the CH3 domains is depicted by the green and black dots. Chain A encompasses an engineered human IgG1 CH2 domain with an engineered human IgG3 CH3 domain. Chain B has engineered human IgG1 CH2 and CH3 domains. CHx, constant domain x; TCR Cα or TCR Cβ, BEAT interface proprietary mutations based on the T cell receptor constant domain α or β, respectively. ISB 2001 BEAT was generated by homology modeling. **b**, Human T cell activation of anti-CD3ε produced as human IgG1 LALA and control isotype by incubating with a dose–response of the cLC Fab bound to the plate. Graph shows mean ± s.d. (*n* = 6 independent T cell donors from two independent experiments). **c**, SPR sensorgrams from a single replicate show blockade of BCMA/APRIL interaction (top sensorgram) and blockade of BCMA/BAFF interaction (bottom sensorgram) upon binding of anti-BCMA-E6 Fab to recombinant human BCMA protein. Curves are colored by anti-BCMA-E6 Fab concentration in BCMA/anti-BCMA premix solution. Data provided are from a single experiment (no repeats). **d**, Competition binding assay by Octet BLI. Curves represent injection of anti-CD38-B3 Fab/daratumumab Fab premix (dashed line) or daratumumab at twofold concentration of saturating solution (solid line) over CD38 immobilized surface saturated with daratumumab Fab from a single replicate in one experiment (no repeats). **e**, Binding sensorgrams of respective representative measurements from three independent replicates show the binding of ISB 2001 to human CD3εδ, human BCMA and human CD38 by SPR. Colored curves represent single concentration injections with serial dilutions of 1:3. Black curves represent 1:1 kinetic fits (BCMA and CD38). For binding to CD3εδ, the *K*_d_ was inferred from a steady-state affinity model. Source data

ISB 2001 consists of anti-CD3ε Fab with a 15-amino-acid flexible glycine–serine peptide linker (3xG_4_S) attached to an anti-BCMA Fab on the BEAT B chain^10,11^ (Fig. 1a). The anti-CD38 Fab is linked to the BEAT A chain. The CD3ε-, BCMA- and CD38-binding domains of ISB 2001 were selected from a synthetic antibody variable heavy (VH) phage display library consisting of VH1-69, VH3-23, VH3-15 and VH3-53 germlines with a Vκ3-15/Jκ1 common light chain (cLC). Hits were identified by library screening against the recombinant ectodomain of the antigen and target-expressing cell lines.

**Extended Data Fig. 1:**
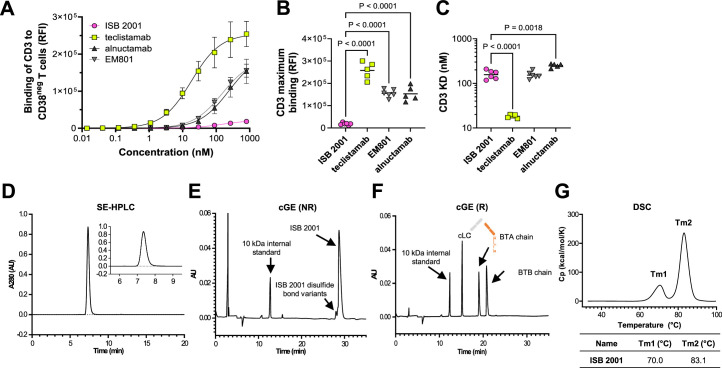
Target binding properties of ISB 2001, alnuctamab, teclistamab and EM801, and biophysical characterization of ISB 2001. **(a)** Curves representing the binding of ISB 2001 to CD38^neg^ T cells compared to teclistamab, EM801 and alnuctamab (n = 6 T cell donors for ISB 2001 and EM801 and n = 5 for teclistamab and alnuctamab performed in 2 independent experiments). Symbols represent the mean ± SD. **(b-c)** Quantification of the maximum binding (RFI) **(b)** and K_D_ (nM**) (c)** to T cells (n = 6 T cell donors for ISB 2001 and n = 5 T cell donors for teclistamab and alnuctamab). Average of maximal binding or Log_10_(K_D_) was compared using repeated measure ANOVA and Dunnett’s multiple comparisons to ISB 2001. Statistical differences are shown in the graphs when significant (P < 0.05). **(d)** Size-Exclusion chromatography profiles of ISB 2001. ISB 2001 eluted mainly as a monodisperse sample (main peak=99.7%) with a retention time of 7.35 min. **(e)** Non-reducing capillary Gel Electrophoresis (cGE (NR)). ISB 2001 accounts for 88.1% of the total species detected. Traces of disulfide bond variants, with one (4%) or two (3.6%) reduced interchain disulfide bonds was observed. The other species detected at smaller molecular weights (ranging from 0.5 to 2% of the total of the species detected), likely arise from artefactual degradation of the molecule in the analysis sample buffer. **(f)** cGE profile in reducing conditions (R) showing three main peaks corresponding to the common light chain (cLC) (37.6%), BEAT chain A (BTA) (26.0%), and BEAT chain B (BTB) (35.0%) at respectively 15.2, 19.0 and 20.8 min, and accounting for 98.6% of the total species detected. **(g)** Thermal stability by Differential Scanning Calorimetry (DSC) shows a first transition at 70.0 °C, corresponding to the melting of ISB 2001 CH2 and CH3 domains, and a second transition at 83.1 °C corresponding to the cooperative unfolding of the different Fabs in ISB 2001. *A280=Absorbance at 280* *nm; AU=Absorbance Units; Tm=melting temperature; Cp=specific heat capacity*. Source data

The anti-CD3ε Fabs were selected for their ability to induce T cell activation (Fig. 1b). The anti-BCMA Fabs were selected to block BCMA–APRIL interaction. Surface plasmon resonance (SPR) shows that anti-BCMA-E6 Fab (nonoptimized variant of anti-BCMA Fab in ISB 2001) effectively blocked the binding of BCMA to both APRIL and BAFF (Fig. 1c). Given that patients will potentially receive daratumumab directly before ISB 2001, the anti-CD38 Fab was selected to avoid binding competition with daratumumab. To evaluate binding properties, competitive biolayer interferometry (BLI) assays were performed. Anti-CD38-B3 Fab (nonoptimized variant of anti-CD38-binding arm of ISB 2001) was able to bind to the CD38 ectodomain in the presence of daratumumab, indicating that both antibodies had different binding epitopes (Fig. 1d). Antibodies underwent affinity maturation by randomization of the heavy chain’s complementarity determining regions (CDRs). ISB 2001 bound to human CD3ε with medium-low affinity (dissociation constant (*K*_d_) = 41.4 nM by SPR) and with high affinity to both BCMA (*K*_d_ = 1.9 nM) and CD38 (*K*_d_ = 2.7 nM) (Fig. 1e and Supplementary Table 1). The affinity of ISB 2001 to CD3ε is comparable to alnuctamab but has a 20-fold faster off-rate and a fivefold lower affinity compared to teclistamab and EM801 (Supplementary Table 1). The affinity of ISB 2001 to CD38-negative T cells is comparable to EM801 and alnuctamab but lower than that of teclistamab (Extended Data Fig. 1a–c). Based on analytical characterization, including size-exclusion high-performance liquid chromatography (SE-HPLC), nonreduced and reduced capillary gel electrophoresis and differential scanning calorimetry (DSC), we demonstrated that ISB 2001 possesses good biophysical properties (Extended Data Fig. 1d–g).

**Extended Data Fig. 2:**
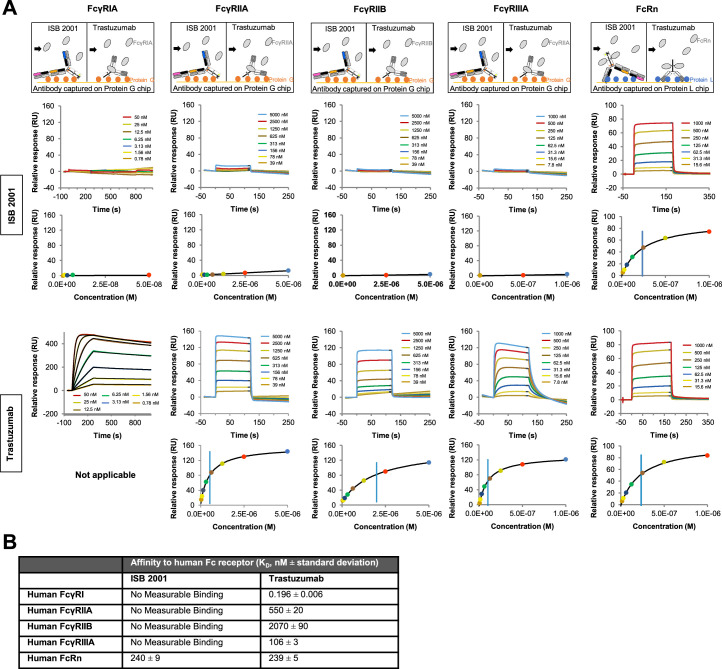
ISB 2001 Binding to Fc receptors by Surface Plasmon Resonance. **(a)** Binding to the FcγR is silenced in ISB 2001. The binding of ISB 2001 to Fcγ receptors Ia, IIa, IIb, IIIa and FcRn was measured by SPR and compared to trastuzumab, a classical Fc-competent IgG1. Cartoons on top show schematic of the respective assay settings. For each receptor and molecule tested, the binding sensorgram and plot of equilibrium response versus concentration, when applicable, of a representative measurement are shown. On the binding sensorgrams, colored lines represent experimental data and black lines represent the fitted data. On the plot of equilibrium response versus concentration, colored dots represent experimental data, black curve represent fitted data and vertical blue bars represent inferred equilibrium dissociation constants (K_D_). For FcγRIA, IIA, IIB and IIIA no or too weak binding of ISB 2001 to Fc receptors was observed to infer a K_D_ value. **(b)** Summary table of equilibrium dissociation constants (K_D_). Data are reported as the average of 3 independent measurements ± SD. Source data

To avoid binding to Fc-γ receptors (FcγRs), the L234A/L235A (LALA)^15^ and P329A^16^ mutations (EU numbering) were introduced into BEAT A and BEAT B heavy chains of ISB 2001, which eliminated binding to FcγRs (Extended Data Fig. 2).

### Avidity and architecture determine ISB 2001 properties

**Fig. 2:**
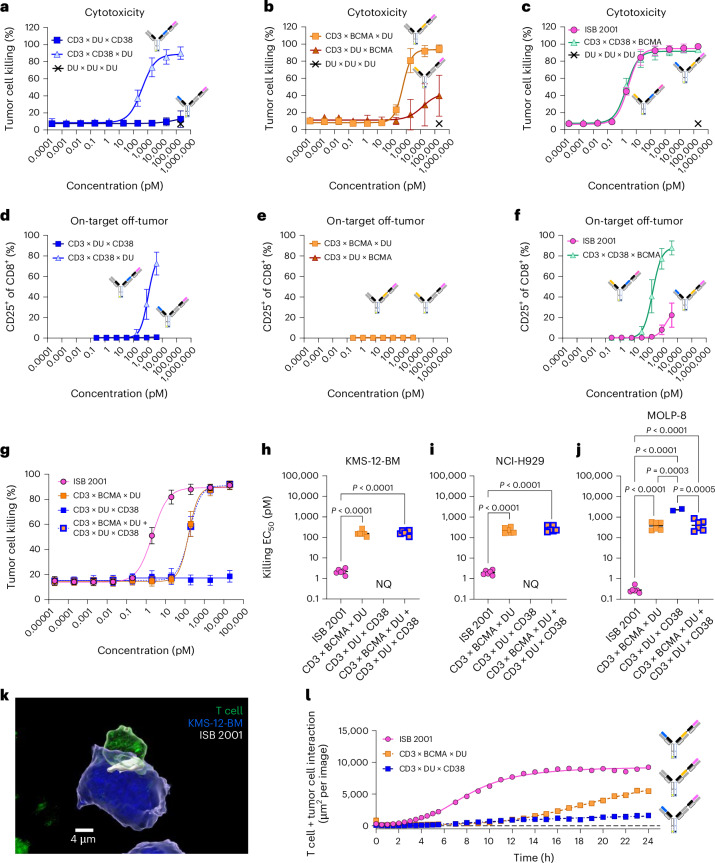
ISB 2001 optimized architecture and avidity binding increases killing and accelerates synapse formation. **a**–**c**, Cytotoxicity of KMS-12-BM cells at different concentrations of CD3 ×DU × CD38 and CD3 × CD38 × DU (**a**), CD3 × BCMA × DU and CD3 × DU × BCMA (**b**) and ISB 2001 and CD3 × CD38 × BCMA (**c**). RDL assays were performed at a 5:1 effector to target ratio for 48 h with purified T cells. Graphs show four-parameter logistic curve fitting with symbols representing mean ± s.d. (*n* = 6 independent T cell donors from two independent experiments). **d**–**f**, T cell activation in a HD-PBMC at different concentrations of CD3 × DU × CD38 and CD3 × CD38 × DU (**d**), CD3 × BCMA × DU and CD3 × DU × BCMA (**e**) and ISB 2001 and CD3 × CD38 × BCMA (**f**). Graphs show four-parameter logistic curve fitting with symbols representing mean ± s.d. (*n* = 6 independent T cell donors from two independent experiments). **g**, Cytotoxicity of the KMS-12-BM cell line at different concentrations of ISB 2001, CD3 × BCMA × DU, CD3 × DU × CD38 and the combination of CD3 × BCMA × DU and CD3 × DU × CD38. Graphs show four-parameter logistic curve fitting with symbols representing mean ± s.d. (*n* = 6 independent T cell donors from three independent experiments). **h**–**j**, EC_50_ values for cytotoxicity on KMS-12-BM (**h**), NCI-H929 (**i**) and MOLP-8 (**j**). log_10_(EC_50_) (*n* = 6 independent T cell donors from three independent experiments; EC_50_ values for CD3 × DU × CD38 were not quantifiable except for *n* = 2 on MOLP-8) were compared using repeated measure ANOVA followed by Tukey’s multiple comparison in **h**–**j**. **k**, Representative confocal image (from three independent experiments) of ISB 2001 (white) and the synapse between a T cell (green) and a KMS-12-BM cell (blue), acquired with a Zeiss LSM 800 inverted confocal microscope, magnification ×40. **l**, Quantification of T cell and KMS-12-BM tumor cell interaction over time using Incucyte live imaging for ISB 2001, CD3 × BCMA × DU and CD3 × DU × CD38. Quantification of T cell and tumor interaction using Incucyte. Graph shows mean of *n* = 6 (ISB 2001) or 5 (CD3 × BCMA × DU and CD3 × DU × CD38) technical replicates from two independent experiments. Statistical differences from post-hoc comparison are shown in the graphs as exact *P* value when statistically significant (*P* < 0.05). NQ, not quantifiable. WT, wild-type. Source data

To assess whether the relative positions of the binding domains impact function, ISB 2001 (CD3 × BCMA × CD38) (Fig. 1a) was compared to CD3 × CD38 × BCMA, in which the anti-CD38 domain is proximal to the anti-CD3ε-domain. Control molecules with one tumor binding domain replaced by an irrelevant dummy Fab (DU) were also produced. The different constructs were first evaluated for tumor killing on a MM cell line in a redirected lysis (RDL) assay. The tumor cytotoxicity of control molecules with single tumor binding domain was superior when the anti-TAA domain was closer to the anti-CD3ε domain (Fig. 2a,b); however, both trispecific molecules showed similar and potent tumor cell cytotoxicity, indicating that either of the two TAAs can be placed in close proximity to the anti-CD3ε domain for maximal cytotoxicity (Fig. 2c).

**Extended Data Fig. 3:**
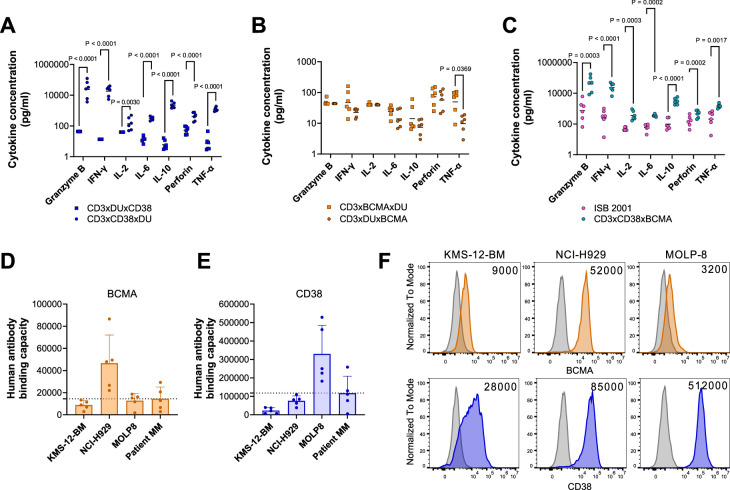
On-target off-tumor T cell cytokine release and characterization of surface target expression on patient samples and MM cell lines. **(a-c)** Quantification of cytokines and cytolytic factors (granzyme B, IFN-γ, IL-2, IL-6, IL-10, perforin and TNF-α) in a high-density PBMC assay comparing the secretion induced by (**a**) CD3 × DU ×CD38 and CD3 × CD38 × DU, (**b**) CD3 × BCMA ×DU and CD3 × DU × BCMA and (**c**) ISB 2001 and CD3 × CD38 × BCMA. Graphs show the maximum secretion in pg/mL. LLOD for each cytokine are the following: granzyme B = 43.4 pg/ml, IFN-γ = 13.7 pg/ml, IL-2 = 39.5 pg/ml, IL-6 = 3.4 pg/ml, IL-10 = 3 pg/ml, perforin = 9.3 pg/ml and TNF-α = 2.9 pg/ml. Each dot corresponds to a different PBMC donor (n = 6 individual PBMC donors from 3 independent experiment). Samples were compared for each cytokines using multiple paired T-Test (two-sided, using false discovery with the two-stage step-up method) and statistical differences are shown in the graphs when statistically significant (P < 0.05). *LLOD=Lower limit of detection* (**d-e**) BCMA and CD38 human antibody binding capacity (BioCytex) on three MM cell lines (KMS-12-BM, NCI-H929 and MOLP-8) and on MM tumor cells from five patients. Each dot represents one patient sample, bars the mean and error bars the SD. The staining on the cell lines were done in parallel to each patient sample. Dotted line corresponds to the mean of all patients tested (**f**) Representative histograms (from 5 repeats) of sABC staining for BCMA (top, orange) and CD38 (bottom, blue) on KMS-12-BM, NCI-H929 and MOLP-8 MM cell lines compared to the isotype antibody (gray). Numbers show the mean of the experiments for specific antigen binding capacity with mouse anti-BCMA and anti-CD38 antibodies followed by anti-mouse FITC-labeled secondary antibody (QIFIKIT). See Supplementary Figure 1 for gating strategy. Source data

As CD38 has broad expression in peripheral blood^17,18^, we hypothesized that the proximity between the anti-CD3ε and anti-CD38 domains may trigger CD38^+^-cell depletion and higher T cell activation in the absence of tumor cells. Indeed, CD3 × CD38 × DU showed higher on-target off-tumor T cell activation and cytokine secretion compared to CD3 × DU × CD38 (Fig. 2d and Extended Data Fig. 3a). Neither CD3 × BCMA × DU nor CD3 × DU × BCMA antibodies demonstrated any detectable on-target off-tumor activity (Fig. 2e and Extended Data Fig. 3b). Notably, the on-target off-tumor T cell activation and cytokine secretion were higher for CD3 × CD38 × BCMA compared to ISB 2001 (Fig. 2f and Extended Data Fig. 3c). These data show that the architecture of ISB 2001 preserves high cytotoxicity toward tumor cells, while inducing minimal T cell activation and cytokine secretion in the absence of tumor cells.

**Extended Data Fig. 4:**
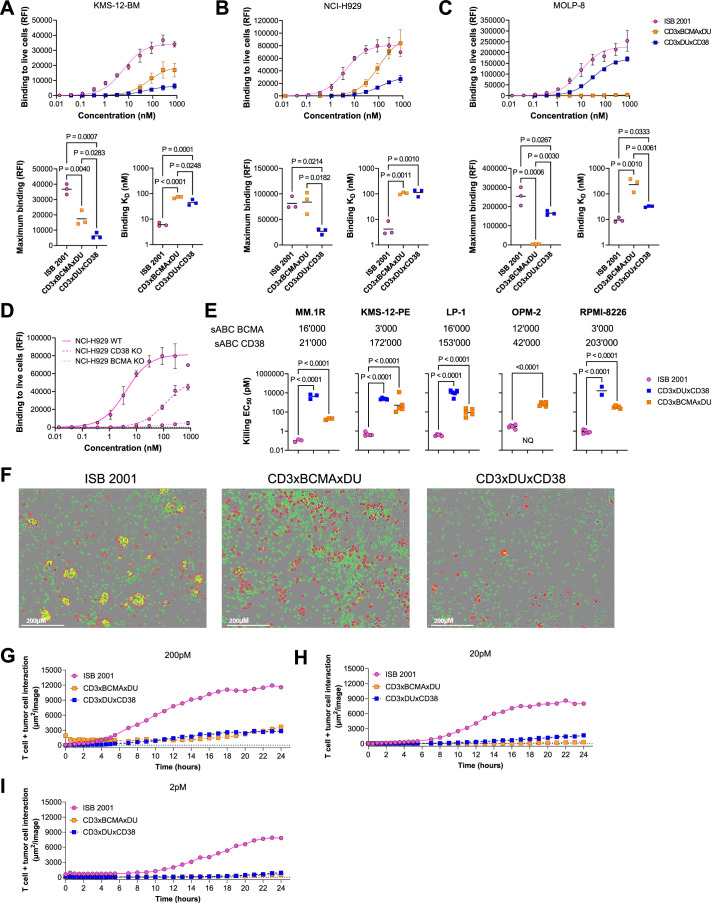
ISB 2001 shows avidity-driven tumor cell binding, killing and synapse formation. (**a-c**) Binding to MM cell lines of ISB 2001 (pink), CD3 × BCMA ×DU (orange) and CD3 ×DUxCD38 (blue). Graphs show 4-parameters logistic curve fitting using variable slope with symbols representing the mean ± SD of 3 experiments (top) and maximum binding and K_D_ for the three molecules are shown (bottom), each dot represents one independent experiment. Log_10_(K_D_) and Max binding of 3 experiments were compared using repeated measure ANOVA model and Tukey HSD comparison. Statistical differences are shown on the graph when statistically significant (P < 0.05) (**d**) Curves showing the binding of ISB 2001 to NCI-H929 cell line expressing CD38 and BCMA (WT, full line), not expressing CD38 (CD38 KO, dashed line) or not expressing BCMA (BCMA KO, dotted line). Graph shows 4-parameters logistic curve fitting using variable slope with symbols representing mean ± SD (n = 3 independent experiments). (**e**) EC_50_ values for cytotoxicity of ISB 2001, CD3xDUxCD38 and CD3 × BCMAxDU on MM cell lines expressing different sABC levels of BCMA and CD38. Log_10_(EC_50_) (n = 6 individual T cell donors from 2 experiments; before exclusion based on acceptance criteria) were compared using repeated measure ANOVA or mixed-effect model (for RPMI-8226 only) followed by Tukey’s multiple comparisons test. **(f**) Representative images captured at 20X of Incucyte data (from 2 independent experiments) from one donor after 18 h of incubation with ISB 2001 (left), CD3 × BCMAxDU (middle) and CD3xDUxCD38 (right), at 2000 pM. Green show T cells, red KMS-12-BM and yellow the interaction between effector and target cells. **(g-i)** Quantification of T cell and KMS-12-BM tumor cell interaction over time using Incucyte live imaging for ISB 2001, CD3 × BCMAxDU and CD3xDUxCD38 at **(g)** 200 pM, **(h)** 20 pM and **(i)** 2 pM. Graphs show mean (n = 6 technical replicates performed on 2 independent experiments using 2 different T cell donors). Source data

ISB 2001 was designed to mediate strong binding to tumor cells expressing low levels of either TAA, through dual targeting of BCMA and CD38. We selected three MM cell lines, KMS-12-BM (BCMA^low^CD38^low^), NCI-H929 (BCMA^int^CD38^int^) and MOLP-8 (BCMA^low^CD38^high^) to model the diversity and heterogeneity in expression of BCMA and CD38 on MM cells (Extended Data Fig. 3d–f and ref. ^19^). ISB 2001 showed higher maximum binding and lower *K*_d_ on the three MM cell lines compared to the CD3 × DU × CD38 and CD3 × BCMA × DU controls (Extended Data Fig. 4a–c). ISB 2001 also showed higher maximal binding to the NCI-H929 wild-type cell line compared to that lacking either BCMA or CD38 (Extended Data Fig. 4d). These data demonstrate the advantage of the avidity binding associated with the dual targeting of BCMA^+^CD38^+^ cancer cells by ISB 2001.

To explore the advantage of avidity binding to trigger cytotoxicity, ISB 2001 was compared to DU controls in RDL assay. ISB 2001 showed significantly increased cytotoxicity against KMS-12-BM cells, compared to the two monotargeted controls, (half-maximum effective concentration (EC_50_) of 2.2 ± 0.7 pM for ISB 2001, not quantifiable for CD3 × DU × CD38 and 164 ± 56 pM for CD3 × BCMA × DU) (Fig. 2g,h). Similar results were observed for NCI-H929 and MOLP-8 cell lines (Fig. 2i, j). Enhanced cytotoxicity of ISB 2001 compared to the monotargeting controls was further demonstrated for five additional cell lines (Extended Data Fig. 4e). The cytotoxic potency of ISB 2001 was also increased compared to the combination of the two controls, suggesting that ISB 2001 activity was driven by avidity binding and not due to the additive effect of targeting two antigens on MM cells independently (Fig. 2g–j). The control molecule CD3 × DU × CD38 showed detectable killing activity mostly when targeting cells with high CD38 expression, usually found in malignancies, such as MOLP-8, RPMI-8226, LP-1 and KMS-12-PE (Fig. 2j and Extended Data Fig. 4e).

TCEs mediate tumor cytotoxicity by forming stable immunological synapses (ISs) between tumor cells and T cells^20,21^. The ability of ISB 2001 to mediate IS formation was evaluated by confocal microscopy. ISB 2001 was enriched at the interface between T and KMS-12-BM cells after 4 h of incubation, suggesting that the trispecific antibody mediated close interactions between tumor and T cells enabling IS formation (Fig. 2k). Live imaging of tumor and T cells incubated with ISB 2001 at 2 nM showed higher contact rate compared to controls (Fig. 2l and Extended Data Fig. 4f). Similar results were observed at lower concentrations of TCEs, although with slower kinetics (Extended Data Fig. 4g–i).

### ISB 2001 confers superior killing than monotargeted TCE

**Fig. 3:**
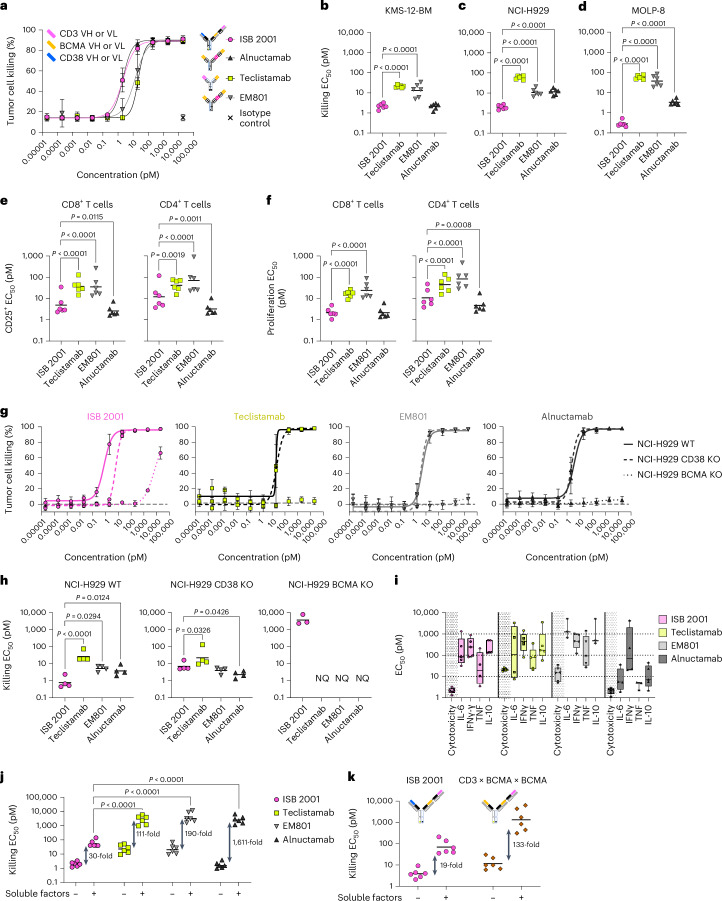
Dual targeting of BCMA and CD38 by ISB 2001 allows efficient tumor cell killing, even in the presence of sBCMA, APRIL and sCD38. **a**–**d**, Cytotoxicity of the KMS-12-BM at different concentrations of ISB 2001, teclistamab, alnuctamab and EM801 (**a**) and EC_50_ values for cytotoxicity of KMS-12-BM (**b**), NCI-H929 (**c**) and MOLP-8 cell lines (**d**). Graph in **a** shows four-parameter logistic curve fitting with symbols representing mean ± s.d. of six independent donors. log_10_(EC_50_) (*n* = 6 independent T cell donors from three independent experiments) were compared using repeated measure ANOVA model and Dunnett’s comparison in **b**–**d**. **e**,**f**, CD8^+^ T cell activation (**e**) and proliferation (**f**) after treatment in an RDL assay against KMS-12-BM. log_10_(EC_50_) (*n* = 6 independent T cell donors from two independent experiments) were compared using repeated measure ANOVA model and Dunnett’s comparison. **g**,**h**, ISB 2001, teclistamab, alnuctamab and EM801 killing curves (**g**) and EC_50_ values for cytotoxicity (**h**) of NCI-H929 WT, CD38^−/−^ and BCMA^−/−^ cell lines. log_10_(EC_50_) (*n* = 4 independent T cell donors (before acceptance criteria exclusion) from two independent experiments) were compared using repeated measure ANOVA model and Dunnett’s comparison to ISB 2001. Graphs in **g** show four-parameter logistic curve fitting with symbols representing mean ± s.d. (*n* = 4 independent T cell donors). **i**, EC_50_ of cytotoxicity, IL-6, IFNγ, TNF and IL-10 release in an RDL assay against KMS-12-BM. Boxplots show 25th to 75th percentile and whiskers minimum and maximum values of *n* = 6 donors from two independent experiments. **j**,**k**, Cytotoxicity in presence (+) or absence (−) of soluble factors after treatment with ISB 2001, teclistamab, alnuctamab and EM801 (**j**) or ISB 2001 and CD3 × BCMA × BCMA molecule (**k**) in an RDL assay. log_10_(EC_50_) (*n* = 6 independent PBMC donors from two experiments) were compared using two-way ANOVA and Tukey’s multiple comparisons in **k** (only differences between ISB 2001 and TCEs with soluble factors are shown). RDL assays were performed at a 5:1 effector to target ratio with purified T cells or PBMCs with six donors for 48 or 72 h. Statistical differences from post-hoc comparison are shown in the graphs as exact *P* value when statistically significant (*P* < 0.05). NQ, not quantifiable. Source data

The cytotoxic potency of ISB 2001 was further compared to TCEs with monovalent or bivalent BCMA targeting: teclistamab^2^, EM801 (ref. ^6^) and alnuctamab^7^. ISB 2001 demonstrated higher cytotoxic potency compared to teclistamab and EM801 on all cell lines and compared to alnuctamab on NCI-H929 and MOLP-8 (Fig. 3a–d). ISB 2001 was able to induce very potent cytotoxicity of MOLP-8 cells, expressing 3,000 BCMA molecules, with 20- to 260-fold lower EC_50_ compared to BCMA-specific TCEs (Fig. 3d). Cytotoxicity correlated with CD8^+^ and CD4^+^ T cell activation and proliferation (Fig. 3e,f).

ISB 2001 showed similar potency to anti-BCMA TCEs on CD38 knockout (KO) NCI-H929 cells but also induced killing of MM cells when BCMA was absent, unlike other TCEs tested (Fig. 3g,h). Despite the high cytotoxic potency of ISB 2001, induction of pro-inflammatory cytokine secretion was similar to the other less-potent TCEs (Fig. 3i), with the cytokine release EC_50_ in the RDL assay ranging from 66 to 356 pM.

**Extended Data Fig. 5:**
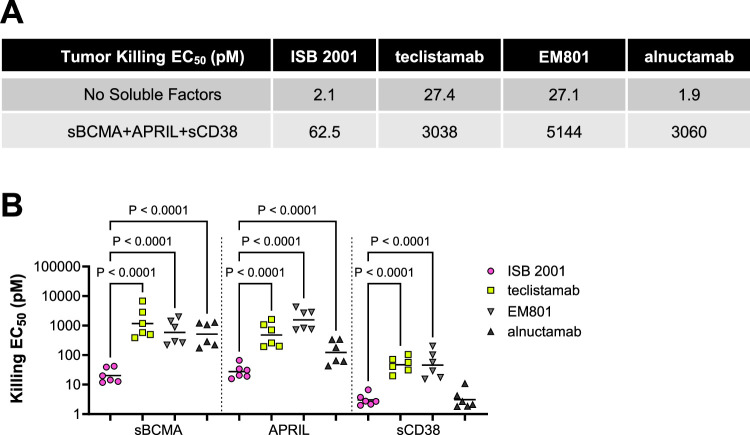
APRIL and sBCMA decrease the killing potency of BCMA TCE and to a lesser extent the potency of ISB 2001. (**a**) Table summarizing average EC_50_ (pM) killing values in the absence or presence of soluble factors (sBCMA, APRIL and sCD38) for ISB 2001, teclistamab, EM801 and alnuctamab in KMS-12-BM cell line. (**b**) EC_50_ killing potency of ISB 2001, teclistamab, EM801 and alnuctamab against the KMS-12-BM cell line in the presence of sBCMA, APRIL or sCD38 alone. PBMC were used as effector cells at a 5:1 ratio.Log_10_(EC_50_) (n = 6 PBMC donors from 2 experiments) were compared using repeated measure ANOVA model and Tukey HSD comparison and statistical differences are shown only to compare ISB 2001 to TCEs for each soluble factors when statistically significant (P < 0.05). Source data

APRIL, sBCMA and sCD38 are found at higher levels in the serum of patients with MM compared to healthy donors (HDs) (up to 50-, 10- and fourfold, respectively)^2,22,23^. These soluble factors either bind to the TCEs in solution (sBCMA and sCD38) or compete with BCMA-targeting TCEs for binding to BCMA on target cells (APRIL). Both interactions interfere with the efficacy of TCEs. ISB 2001 and the BCMA-targeting TCEs were tested in the presence of soluble factors at similar level to those found in patients with MM. The cytotoxic potency of all tested antibodies was decreased (Fig. 3j); however, ISB 2001 was less affected by the combination of sBCMA, APRIL and sCD38 than the other TCEs (Fig. 3j and Extended Data Fig. 5a). APRIL and sBCMA affected all BCMA-targeting TCEs when assessed separately and the effect was additive for the combination of APRIL and sBCMA, whereas, no effect was observed with sCD38 alone (Extended Data Fig. 5b).

To further understand the ISB 2001 resistance to soluble factors, ISB 2001 was compared to a bivalent control (CD3 × BCMA × BCMA). The bivalent control showed lower cytotoxic potency compared to ISB 2001. Soluble factors affected its killing capacity by 133-fold compared to 19-fold for ISB 2001 (Fig. 3k), demonstrating that monovalent targeting of two TAAs by a TCE is superior to bivalent targeting of a single TAA in the context of competing soluble factors.

### ISB 2001 shows a favorable safety profile

**Fig. 4:**
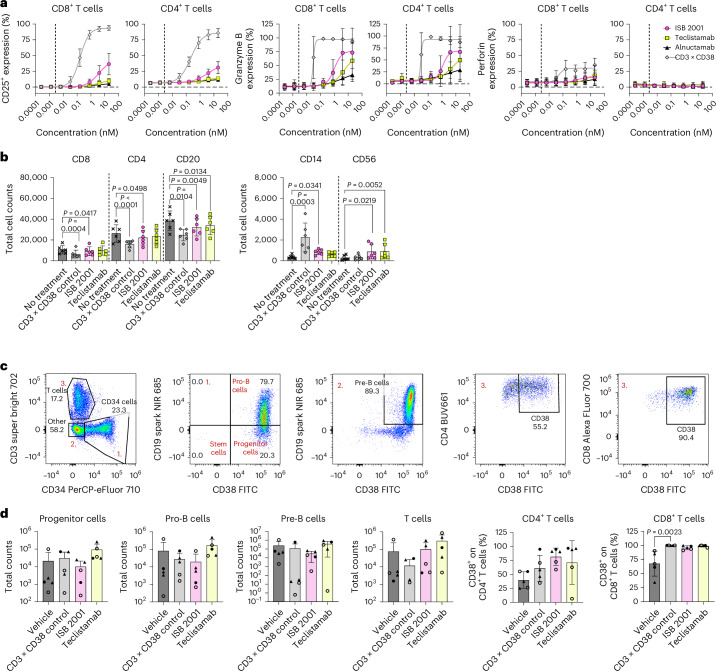
ISB 2001 has a tolerability profile comparable to teclistamab both in vitro and in vivo. **a**, T cell activation and cytolytic molecules secretion in HD-PBMC assay treated with ISB 2001, teclistamab, alnuctamab and CD3 × CD38 control. Percentage of CD25^+^ (left), granzyme B (middle) and perforin (right) of CD8^+^ and CD4^+^ T cells are shown in a four-parameter logistic curve fitting with symbols representing mean ± s.d. (*n* = 6 independent PBMC donors from two independent experiments for CD25^+^; *n* = 3 independent PBMC donors from two independent experiments for granzyme B and perforin). Dashed line represents the EC_90_ of ISB 2001 killing potency in RDL assay on KMS-12-BM. **b**, PBMC counts in HD-PBMC assay treated with 1 nM of ISB 2001, teclistamab, alnuctamab and CD3 × CD38 control. Total counts of CD8^+^, CD4^+^ and CD20^+^ (left) and total counts of CD14^+^ and CD56^+^ from live cells (right). Cell counts were compared using RM ANOVA (CD4^+^ and CD20^+^) or Friedman test (CD8^+^ and CD56^+^), when donor pairing was effective or Kruskal–Wallis (CD14^+^) test when it was not. Multiple comparisons were performed using Tukey (following RM ANOVA) or Dunn’s multiple comparisons (following Friedman or Kruskal–Wallis). Bars represent the mean and error bars the s.d. (*n* = 6 independent PBMC donors from two independent experiments). **c**,**d**, Quantification of CD38-expressing cells (progenitors, B cell committed progenitors and mature T cells) in the bone marrow of HIS-NXG mice 3 days after treatment with 1.5 mg kg^−1^ ISB 2001, teclistamab, CD3 × CD38 control or vehicle control (*n* = 5 bone marrow samples from independent mice from one experiment). Gating of cell populations used for the plots in **d** from one vehicle mouse (**c**). Red numbers located in the top left corner of the last four dot plots refer to the gated populations shown in the first dot plot. Plots summarizing the total count and percentage of CD38^+^ cells of the indicated populations in the bone marrow (**d**). Mice reconstituted with different CD34^+^ donor cells are represented with different symbols; bars represent the mean and error bars the s.d. (*n* = 5 bone-marrow samples from independent mice from one experiment). Samples were compared using Friedman test or one-way ANOVA (only for CD4^+^ T cells) followed by a Dunn’s multiple comparisons. Differences are shown in the graphs as exact *P* value when statistically significant (*P* < 0.05). Source data

The expression of CD38 on some peripheral blood cell populations could potentially limit the therapeutic window of ISB 2001. Thus, the on-target off-tumor activity of ISB 2001 was compared to a CD3 × CD38 TCE control and to the CD3 × BCMA TCEs teclistamab and alnuctamab in an HD peripheral blood mononuclear cell (PBMC) assay. In the absence of tumor cells, ISB 2001 exhibited only minor T cell activation (as measured by CD25 upregulation on CD8^+^ and CD4^+^ T cells) compared to the CD3 × CD38 TCE control. The induction of granzyme B in CD8^+^ and CD4^+^ T cells and perforin in CD8^+^ T cells was also lower for ISB 2001 compared to the CD3 × CD38 TCE (Fig. 4a). At very high concentration (>1 nM), T cell activation and granzyme B induced by ISB 2001 were slightly elevated compared to BCMA-targeting TCEs (Fig. 4a). Nevertheless, this concentration is around 400-fold higher than the concentration at which ISB 2001 reaches the EC_90_ in an RDL assay (2.7 ± 1.3 pM), suggesting sufficient therapeutic window for this molecule. This difference in T cell activation did not contribute to the additional depletion of different cell populations, as similar number of CD4^+^ T cells, CD8^+^ T cells, CD20^+^ B cells, CD14^+^ monocytes and CD56^+^ natural killer (NK) cells were recovered at the end of the HD-PBMC assay following treatment with teclistamab or ISB 2001 (Fig. 4b).

Finally, to test whether ISB 2001 has any on-target off-tumor activity in vivo, we quantified the number of hematopoietic progenitors, B cell-committed progenitors and mature T cells present in the bone marrow of CD34^+^ humanized NXG mice 3 days after treatment with ISB 2001 (1.5 mg kg^−1^). TCEs targeting either BCMA (teclistamab) or CD38 (CD3 × CD38 control molecule) were used as controls at the same dosage of 1.5 mg kg^−1^. The counts of hematopoietic progenitor cells, pro-B cells, pre-B cells and T cells in the bone marrow of ISB 2001-treated mice were similar to the vehicle-treated mice (Fig. 4c,d), showing no depletion of CD38-expressing cells by ISB 2001. Of note, all TCEs showed an increase in CD38 expression on T cells, consistent with some immune activation resulting from the therapy. The CD38-expressing progenitors were present and were not killed by ISB 2001 or control CD38 or BCMA-targeted TCEs.

### ISB 2001 outperforms a combination of BCMA and CD38 therapies

**Fig. 5:**
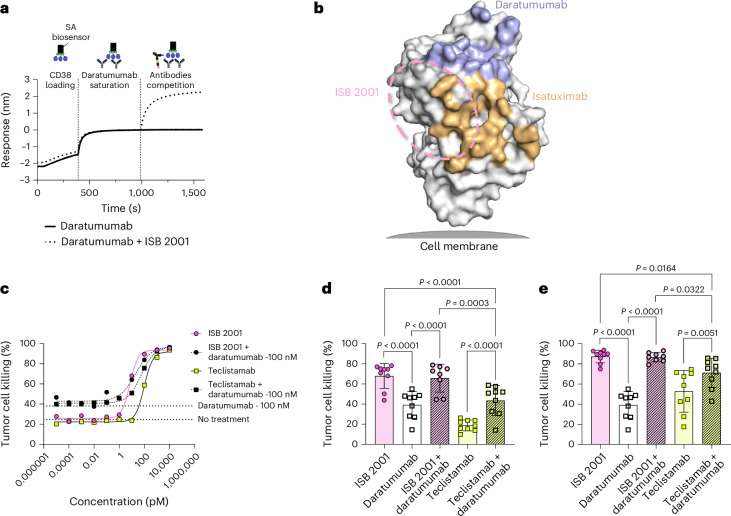
ISB 2001 cytotoxic potency is not affected by daratumumab co-treatment and shows stronger killing potency than the combination of a BCMA TCE and daratumumab. **a**, Competition binding assay by Octet BLI shows the absence of competition between ISB 2001 and daratumumab, as suggested by additive binding signal upon sequential exposure of a daratumumab-saturated CD38 sensor surface to ISB 2001 (dotted line). Saturation of the CD38 sensor surface was verified by dipping the daratumumab-saturated CD38 sensor surface into a solution of daratumumab at twofold concentration of saturation solution (solid line). Representative plot shows binding to the sensor tip as a wavelength shift (response) versus time (*n* = 2 independent measurements). **b**, Surface representation of CD38 illustrating the hypothetical epitope bin of ISB 2001 (dashed line), as determined from epitope binning assays including daratumumab and isatuximab. The epitopes of daratumumab (PDB 7DHA) and isatuximab (PDB 4CMH) are colored blue and orange, respectively. **c**, Cytotoxicity of the KMS-12-BM cell line at different concentrations of ISB 2001 and teclistamab in the presence or absence of 100 nM daratumumab in a MMoAK assay. Dotted lines show the no-treatment and daratumumab at 100 nM-only conditions. Four-parameter logistic curve (**c**) fitting from a representative donor (*n* = 9 individual PBMC donors and *n* = 8 individual donors for ISB 2001 + daratumumab, from *n* = 3 independent experiments). **d**,**e**, Cytotoxicity of ISB 2001 and teclistamab at 10 pM (**d**) or 100 pM (**e**) daratumumab (at 100 nM) and a combination of ISB 2001 or teclistamab (at 10 and 100 pM, respectively) plus daratumumab (at 100 nM) in a MMoAK assay. Each dot represents one individual donor (*n* = 9 or *n* = 8 for ISB 2001 + daratumumab) and bars show the mean ± s.d. from four independent experiments. Means were compared using a mixed-effects model followed by a Tukey’s multiple comparison and statistical differences are shown as exact *P* value when statistically significant (*P* < 0.05). Source data

Daratumumab levels in patients’ circulation remain high for a few weeks after treatment^24^ which may affect the binding and cytotoxicity of CD38-targeted therapies. As expected, based on the selection criteria of the parental anti-CD38 domain, BLI confirmed that the affinity-matured anti-CD38 Fab domain in ISB 2001 does not compete with daratumumab (Figs. 1d and 5a). The putative binding site of ISB 2001 on CD38, determined by epitope binning, is shown as an ellipse on CD38 (Fig. 5b). The cytotoxicity of ISB 2001 in the presence or absence of daratumumab was measured in a multiple mode of action killing (MMoAK) assay. This assay measures target killing induced by T cells in RDL, antibody-dependent cell-mediated cytotoxicity antibody-dependent cellular phagocytosis and complement-dependent cytotoxicity. Daratumumab, which has maximal cytotoxicity of 36 ± 18.5% at 100 nM, did not interfere with the cytotoxicity of ISB 2001, suggesting that it could be used immediately after patients’ relapse from daratumumab (Fig. 5c–e).

In vitro, the addition of daratumumab to teclistamab increased the maximal cytotoxicity of teclistamab by 1.3–2.3-fold (Fig. 5d,e). Nevertheless, ISB 2001 treatment at 10 and 100 pM induced higher tumor cell killing relative to the combination of teclistamab (10 or 100 pM) and daratumumab at 100 nM (Fig. 5d,e).

### ISB 2001 induces killing of tumor cells from MM patients

**Fig. 6:**
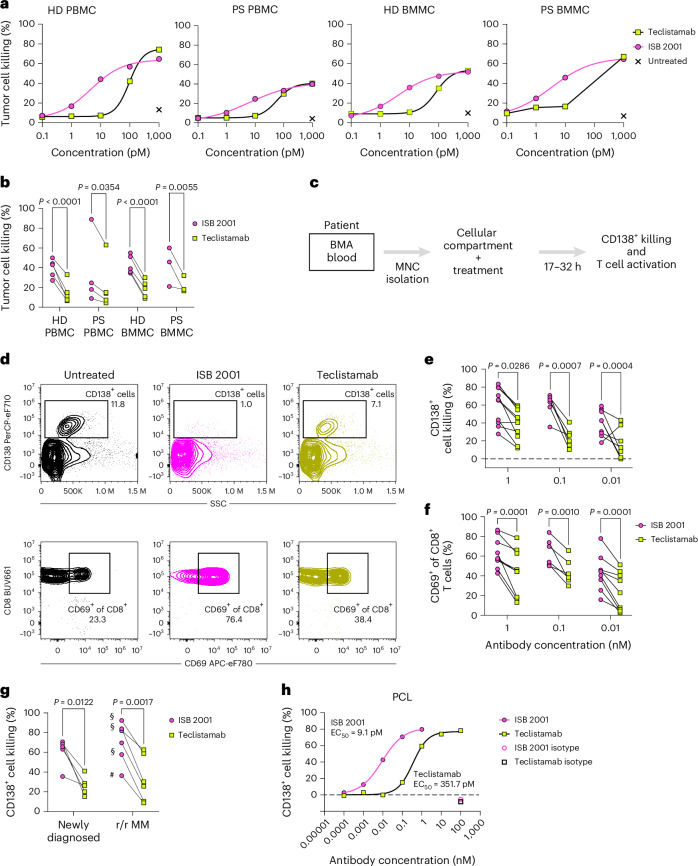
ISB 2001 maintains potency to kill primary tumor cells from patients with MM. **a**,**b**, Representative curves of cytotoxicity of KMS-12-BM cell line at different concentrations of ISB 2001 and teclistamab by T cells isolated from HD- or PS-PBMCs (*n* = 5 and *n* = 2, respectively) or BMA (*n* = 5 and *n* = 2 donors, respectively) (**a**) and cytotoxicity at 10 pM (**b**). Percentage of cytotoxicity of *n* = 3 (PS-BMMC donors) or *n* = 5 (HD- and PS-PBMC and HD-BMMC donors, before acceptance criteria application) from *n* = 5 independent experiments were compared using REML followed by Šidák’s multiple comparison for each population of T cells. **c**, Experimental setup schema to assess ISB 2001 and teclistamab cytotoxic capacity of CD138^+^ tumor cells in BMA and T cell activation. **d**, Representative dot plots of CD138^+^ cell killing (top) and CD69^+^ of CD8^+^ T cells (bottom). **e**,**f**, Cytotoxicity of CD138^+^ tumor cells (**e**) or T cell activation (CD69^+^) (**f**) on samples from patients with MM treated with ISB 2001 or teclistamab at 0.01 (*n* = 10 PS for cytotoxicity and *n* = 9 PS for T cell activation), 0.1 (*n* = 6 PS for cytotoxicity and T cell activation) and 1 nM (*n* = 10 PS for cytotoxicity and *n* = 9 PS for T cell activation). CD138^+^ cell killing and T cell activation were compared using REML followed by Šidák’s multiple comparison for each concentration. **g**, Cytotoxicity of CD138^+^ tumor cells on samples from newly diagnosed (left, *n* = 4 PS) or patients with r/r MM (right, *n* = 6 PS), treated with ISB 2001 or teclistamab at 0.1 nM. Graph shows dots for individual samples. Previous treatments are stated in the graph as § for anti-CD38-treated or # for anti-BCMA-treated PS. Percentage of CD138^+^ cell killing was compared using a Holm–Šidák’s multiple two-sided paired *t*-test. **h**, Cytotoxicity curve of CD138^+^ MM cells by ISB 2001, teclistamab and isotype controls at 20 h in a sample from PCL (*n* = 1 PS). Graph shows four-parameter logistic curve fitting and symbols represent the mean of replicates (*n* = 2 replicates for ISB 2001 and teclistamab and *n* = 4 replicates for isotype controls). PCL, plasma cell leukemia. Statistical differences are shown in graphs as exact *P* value when statistically significant (*P* < 0.05). Source data

The cytotoxicity of TCEs in patients with MM may be influenced by the tumor microenvironment and by the number and functional state of effector T cells^25^. The capacity of ISB 2001 to induce MM cell killing was evaluated in a RDL assay employing T cells isolated either from HDs or patients with MM, using PBMCs or bone-marrow mononuclear cells (BMMCs). ISB 2001 and teclistamab killed KMS-12 BM cells independently of T cell origin; however, ISB 2001 consistently showed superior killing (Fig. 6a,b).

The cytotoxicity and T cell activation induced by ISB 2001 were further assessed using bone marrow aspirate (BMA) from patients with MM. The cytotoxicity and T cell activation induced by ISB 2001 and teclistamab were measured following the addition of antibodies to the patient cells for 17–120 h (Fig. 6c). ISB 2001 demonstrated superior cytotoxicity and activation of T cells compared to teclistamab at 1, 0.1 and 0.01 nM in both newly diagnosed and r/r MM patient-derived BMA (Fig. 6d–g). Notably, we evaluated one patient, who relapsed after elranatamab treatment, a BCMA-specific TCE (Fig. 6g). While teclistamab induced less than 10% cytotoxicity, ISB 2001 induced fourfold superior cytotoxicity likely due to overcoming BCMA-downregulation mechanisms.

ISB 2001 was also compared to teclistamab in one blood sample from a patient with plasma cell leukemia, an aggressive disease characterized by high numbers of plasma cells in the peripheral blood. Again, ISB 2001 showed superior cytotoxicity compared to teclistamab (Fig. 6h).

**Extended Data Fig. 6:**
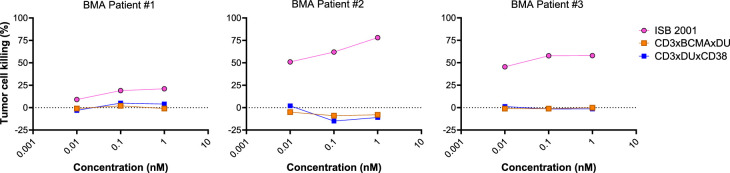
ISB 2001 tumor cell killing of bone marrow aspirates is driven by the avidity effect. Tumor cell killing of bone marrow aspirates (BMA) of ISB 2001 (pink), CD3 × BCMA ×DU (orange) and CD3 ×DU ×CD38 (blue). Graphs show three Multiple Myeloma patients performed in 3 independent experiments. Each dot represents one dose for each molecule. Source data

The importance of the avidity effect of targeting two TAA was also demonstrated in a cytotoxicity assay with BMA from patients with MM (Extended Data Fig. 6). Indeed, we could demonstrate that ISB 2001 was able to induce killing of tumor cells from the bone marrow of patients with MM at different concentrations, whereas the control molecules lacking either the anti-CD38 or the anti-BCMA binding domain could not.

### Tumor regression in xenograft mouse models

**Fig. 7:**
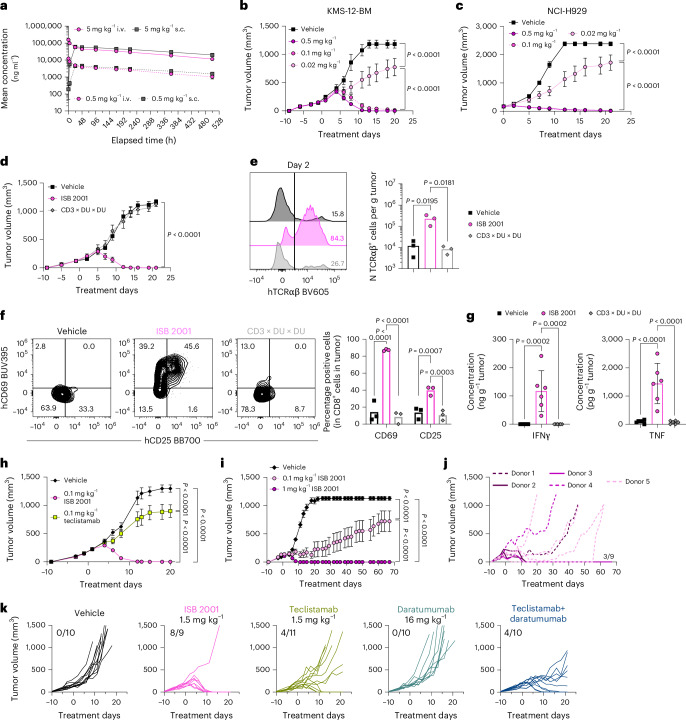
ISB 2001 shows superior antitumor activity to BCMA TCE benchmarks in xenograft mouse models. **a**, ISB 2001 plasma concentration over time in NCG mice following single i.v. and s.c. injection (*n* = 4 mice per group). **b**–**d**, NSG or NCG mice were engrafted s.c. with KMS-12-BM or NCI-H929 cells, respectively and inoculated i.p. with PBMCs. Tumor volume is shown following treatment of KMS-12-BM (*n* = 8 mice per group, except vehicle *n* = 7) (**b**) or NCI-H929 (*n* = 9 mice per group) with ISB 2001 (**c**) and following treatment of KMS-12-BM with ISB 2001 or CD3 × DU × DU at 0.5 mg kg^−1^ (*n* = 8 mice per group) (**d**). Mean ± s.e.m. are shown. Tumor volumes were compared using two-way ANOVA with Tukey’s multiple comparisons in **b**–**d**. **e**,**f**, KMS-12-BM tumors were analyzed ex vivo 2 days after treatments (*n* = 3 mice/group). Expression of human TCRαβ in human CD45^+^ cells and number of TCRαβ cells per gram of tumor (bars represent the mean and samples were compared using one-way ANOVA followed by Tukey’s multiple comparisons) (**e**). Contour plots show the expression of human CD69 versus human CD25 in tumor infiltrating CD8^+^ T cells and graphs show the percentage of both markers in CD8^+^ T cells (bars represent the mean and samples were compared using two-way ANOVA followed by uncorrected Fisher’s LSD multiple comparisons) (**f**). **g**, Concentration of cytokines in NCI-H929 tumor supernatant (*n* = 6 mice per group, bars represent the mean ± s.d. and samples were compared using one-way ANOVA followed by uncorrected Fisher’s LSD multiple comparisons). **h**–**k**, KMS-12-BM tumor volume following treatment in NSG tumor-bearing mice inoculated with PBMCs from healthy human donors (*n* = 8 mice per group, except for teclistamab where *n* = 7) (**h**) and HIS-NXG mice (*n* = 9 mice per group, except for vehicle where *n* = 12) pretreated with 200 mg kg^−1^ of IVIG (**i**–**k**). Mean ± s.e.m. are shown (**h**,**i**). Tumor volumes were compared using a two-way ANOVA with Tukey’s multiple comparisons. Tumor growth of individual mice shown in **i** (0.1 mg kg^−1^ ISB 2001 group) (**j**). Tumor growth of individual mice upon treatment with the indicated molecules (**k**). Each line represents an individual mouse (**j**,**k**). Number of mice rejecting the tumor is indicated on the graphs. Statistical differences are shown in graphs as exact *P* value when statistically significant (*P* < 0.05). Source data

The pharmacokinetic (PK) parameters of ISB 2001 were determined following single-dose intravenous (i.v.) and subcutaneous (s.c.) administrations in immunodeficient NCG mice. The PK profile of ISB 2001 was dose-linear and demonstrated a greater than 1 week elimination half-life (*t*_1/2_). The mean *t*_1/2_ ranged from 8.0 days to 11.0 days following i.v. and s.c. administrations with excellent s.c. bioavailability of roughly 135% (Fig. 7a), supporting weekly dosing regimen used for subsequent efficacy studies in xenograft models.

**Extended Data Fig. 7:**
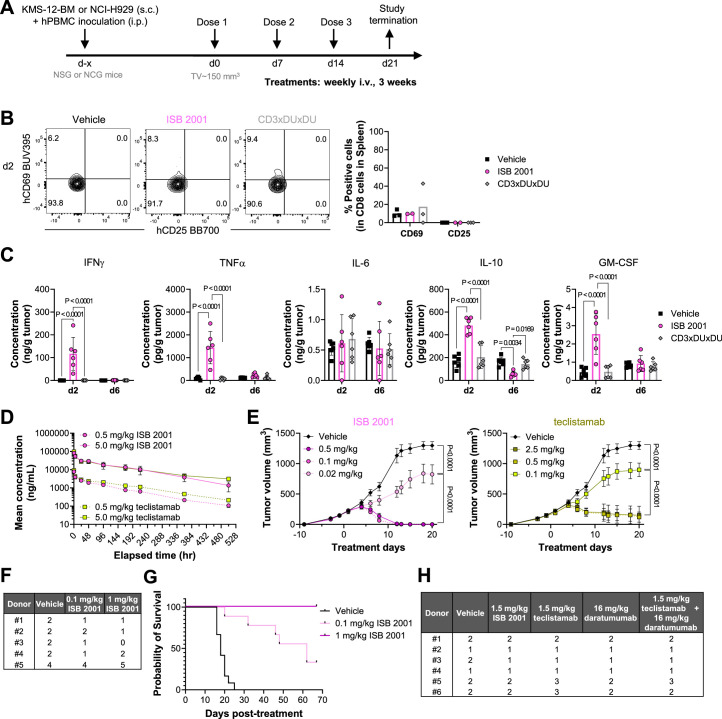
ISB 2001 shows superior antitumor activity to BCMA TCE benchmarks in xenograft mouse models. **(a)** Schematic representation of KMS-12-BM and NCI-H929 hPBMC xenograft mouse models. **(b)** KMS-12-BM tumors of ISB 2001 or CD3xDUxDU (0.5 mg/kg) treated mice were analyzed ex vivo (n = 3 mice/group, except for ISB 2001 where n = 2 from 1 experiment using 2 PBMC donors). Contour plots show the expression of human CD69 vs human CD25 in splenic CD8^+^ T cells and graphs show the percentage of human CD69 (left) and human CD25 (right) positive CD8^+^ T cells in the spleen on day 2 (bars represent the mean). See Supplementary Figure 2 for gating strategy. **(c)** Graphs display the concentration of the indicated cytokine in the NCI-H929 tumor supernatant normalized per gram of tumor at day 2 or 6 post single dose (bars represent the mean and error bars the SD, n = 6 from 1 experiment using 2 PBMC donors). **(d)** NCG mice pretreated with 200 mg/kg of Intravenous Immunoglobulin (IVIG) were injected i.v. with two concentrations (0.5 or 5 mg/kg) of ISB 2001 or teclistamab and monitored for the presence of antibody in plasma at various time points. Graph depicts mean concentration ± SD (ng/mL) vs time of sample collection (n = 4 mice/group,1 experiment). **(e)** KMS-12-BM tumor volume (in mm^3^) is shown following treatment with ISB 2001 (left) or teclistamab (right) at the indicated concentrations in NSG tumor-bearing mice inoculated with PBMCs from healthy human donors and pretreated with 200 mg/kg of IVIG. Each dot represents the average of 8 mice/group (except for teclistamab at 0.5 and 0.1 mg/kg where n = 7) and error bars represent the SEM with Last Observation Carried Forward (LOCF). Differences in tumor volume were determined using a two-way ANOVA with Tukey’s multiple comparisons test and differences are shown in the graphs when statistically significant (P < 0.05). **(f)** Table summarizing the distribution of the CD34-reconstituted mice shown in Fig. 7i, j. **(g)** Graphs is depicting the survival of the mice shown in Fig. 7i, j (n = 9 mice/group, except for vehicle where n = 12 mice/group). **(h)** Table summarizing the distribution of the CD34-reconstituted mice shown in Fig. 7k. Source data

The in vivo activity of ISB 2001 was then evaluated in an MM xenograft model, in which NSG mice were inoculated subcutaneously with KMS-12-BM tumor cells (BCMA^low^CD38^low^) and human PBMCs from HDs (Extended Data Fig. 7a). Following ISB 2001 treatment, significant tumor growth control was observed at all doses tested (0.5, 0.1 and 0.02 mg kg^−1^) compared to vehicle (Fig. 7b). Similar results were obtained in an NCI-H929 (BCMA^int^CD38^int^) xenograft model, in which complete tumor regression was observed at 0.1 mg kg^−1^ ISB 2001 (Fig. 7c). Taken together, these data show a strong, dose-dependent, efficacy of ISB 2001. Moreover, tumor regression was TAA-specific: ISB 2001 at 0.5 mg kg^−1^ induced complete regression of established KMS-12-BM, whereas a control molecule targeting only CD3ε (CD3 × DU × DU) had no effect at the same dose (Fig. 7d).

On day 2, tumor infiltrating CD8^+^ T cells had an activated phenotype showed by the elevated levels of CD69^+^ and CD25^+^ on T cells in ISB 2001-treated mice relative to both vehicle and CD3 × DU × DU (Fig. 7e,f), which was further supported by the elevated levels of effector cytokines (IFNγ and TNF) measured in the tumor supernatants (Fig. 7g). No systemic activation was observed (Extended Data Fig. 7b). No or very low levels of other cytokines found post-TCE treatment such as interleukin (IL)-6 and IL-10 were detected in the tumor supernatant (Extended Data Fig. 7c).

The in vivo potency comparison between ISB 2001 and teclistamab was evaluated in a KMS-12-BM PBMC transfer xenografted model. ISB 2001 induced an antitumor response in all animals at higher doses (0.1 and 0.5 mg kg^−1^). Partial efficacy was observed at the lowest dose (0.02 mg kg^−1^), as well as a reduction in half-life (*t*_1/2_ = 4.3 days), likely caused by competition for mouse FcRn. In contrast, teclistamab, with dosing based on a previous study^2^ (0.1, 0.5 and 2.5 mg kg^−1^), showed lower overall efficacy despite having slightly superior PK (*t*_1/2_ = 5.8 days) in this model (Extended Data Fig. 7d,e). At 0.1 mg kg^−1^ ISB 2001 induced complete tumor regression in 100% of mice (8 out of 8 mice), whereas teclistamab showed only partial responses (30.8% of tumor growth inhibition, 0 out of 7 complete regressions) (Fig. 7h). These data suggest that simultaneous targeting of BCMA and CD38 can lead to an increased cytotoxicity and tumor clearance in vivo.

To assess the long-term impact of treatment, ISB 2001 was evaluated in mice engrafted with human CD34^+^ cells. All mice treated with 1 mg kg^−1^ of ISB 2001 experienced complete tumor regression. While all mice treated with 0.1 mg kg^−1^ ISB 2001 responded, only 4 out of 9 mice rejected tumors (Fig. 7i,j and Extended Data Fig. 7f,g). After 3 weeks of treatment, mice continued to be monitored for 46 days and all mice with palpable tumors experienced tumor regrowth albeit with greatly reduced kinetics compared to vehicle-treated mice (Fig. 7j).

Finally, the combination of daratumumab with teclistamab was compared to ISB 2001 using the route of administration and dosage under clinical evaluation^26^. While only 4 out of 11 mice rejected the tumor upon teclistamab treatment (Fig. 7k), ISB 2001 led to tumor rejection in 8 out of 9 mice, with one nonresponder. Daratumumab as a single agent showed no impact on tumor growth but improved the efficacy of teclistamab treatment. All mice responded to the combination, but only 4 out of 10 mice rejected the tumor, similarly to teclistamab alone.

### QSP modeling allows MPAD prediction of ISB 2001 starting dose

**Fig. 8:**
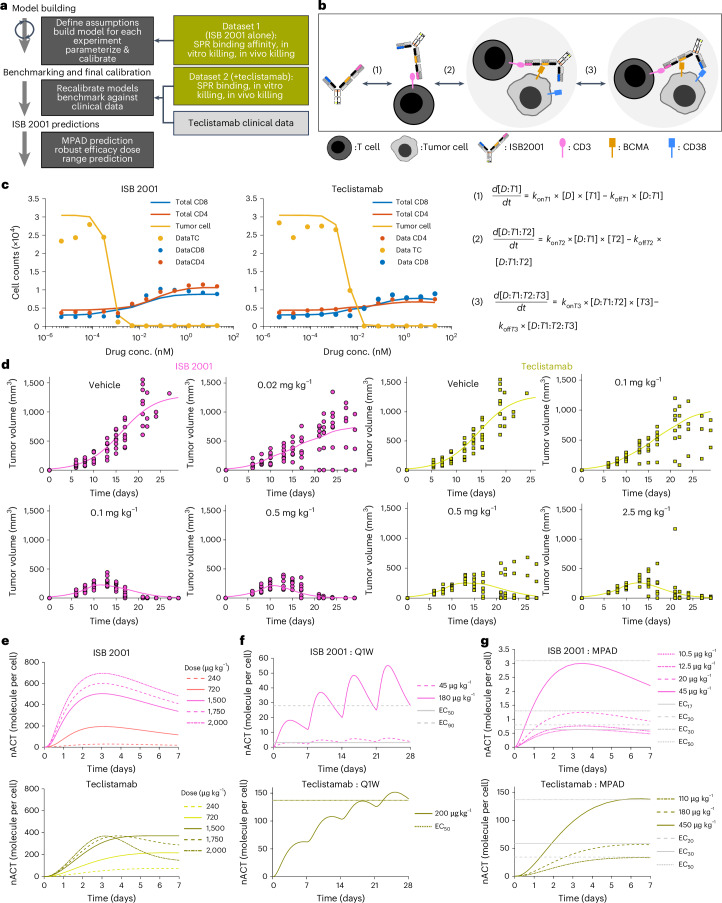
Human QSP modeling. **a**, Workflow for developing QSP model. **b**, Simplified binding schematic with accompanying equations below, where [*D*] is ISB 2001 concentration; [*T*1], [*T*2] and [*T*3] are the concentrations of free CD3, BCMA and CD38; [*D*:*T*1] is a concentration of dimer complex of ISB 2001-CD3; [*D*:*T*1:*T*2] is a concentration of trimer complex of ISB 2001–CD3–BCMA; [*D*:*T*1:*T*2:*T*3] is a concentration of tetramer complex of ISB 2001–CD3–BCMA–CD38; *k*_on_*T*1, *k*_on_*T*2 and *k*_on_*T*3 are the association rate constants for CD3, BCMA and CD38, respectively; and *k*_off_*T*1, *k*_off_*T*2 and *k*_off_*T*3 are the dissociation rate constants for CD3, BCMA and CD38, respectively. **c**,**d**, Goodness-of-fit plots for ISB 2001 and teclistamab dose–response data in vitro and in vivo post-calibration of killing models. RSE for in vitro (CD8, CD4 and tumor) ISB 2001 (30%, 29% and 12%) and teclistamab (20%, 33% and 10%). RSE in vivo ISB 2001 4.7% teclistamab 9.9%. Symbols represent individual experimental data points (tumor, CD4^+^ T and CD8^+^ T cell counts for *n* = 1 representative donor out of 6 (**c**) and tumor volume for *n* = 8 mice per group (**d**)). Lines show model simulations. **e**, nACT plots showing maximum nACT for a range of doses. **f**, Repeated dose nACT levels reach the EC_50_ and EC_90_ thresholds (ISB 2001) and EC_50_ threshold for teclistamab. **g**, MPAD predictions for ISB 2001 and teclistamab. All RSE values are calculated at the EC_50_ of the effect modeled*.* Graphs in **e**–**g** show model predictions of nACT levels in simulated patients. Source data

To establish the optimal FIH dose for ISB 2001 in the absence of cross-reactivity to toxicology species a QSP model was developed to link TCE mechanism of action to predicted patient outcome^27–32^ (Fig. 8a and Methods).

**Extended Data Fig. 8:**
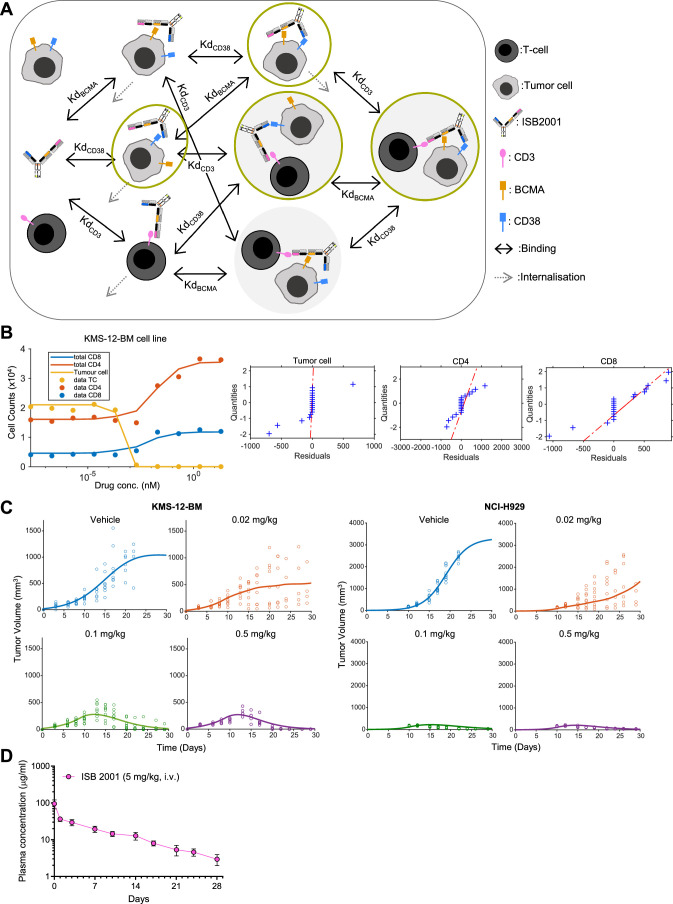
Human QSP modeling. (**a**) Full Schematic of binding model (in vitro). Green circles show species with CD38 binding not found with teclistamab. (**b**) Goodness of fit plot for in vitro parameterization. RSE CD8-30%, CD4-29%, Tumor-12% Residual distribution plots for tumor, CD4 and CD8 T cells (**c**) Goodness of fit plot for in vivo parameterization. RSE KMS-116%, NCI-35% (**d**) TG32 SCID single-dose PK. (5 mg/kg). Source data

The model building was an iterative process, using the output of earlier models to determine key parameters for subsequent models. First, a binding model was created to describe the formation of pharmacologically active species (TCE-mediated trimers/tetramers). These interactions are described using equilibrium binding kinetics (Fig. 8b and Extended Data Fig. 8a). This model enabled calculation of the normalized activation (nACT), indicating how many pharmacologically active trimers/tetramers are present per tumor cell. Second, the in vitro pharmacology model linked cytotoxicity, T cell proliferation and activation to nACT. The model was evaluated by goodness-of-fit diagnostic plots at the experiment end with experimental data falling close to the modeled line, confirming a good fit (Extended Data Fig. 8b). Third, a modified minimal physiologically based PK (PBPK) model^33^, including a tumor compartment was created. Then a PK/pharmacodynamic (PKPD) model was built to link drug concentration to nACT during tumor regression. The goodness of fit was assessed (Extended Data Fig. 8c) showing that the model described the data adequately, though variable tumor growth at some concentrations could not be captured. Finally, a human PBPK model was developed using parameters scaled from hFcRn Tg32 SCID mice, which have been shown to be predictive of human clearance of antibodies^34^ (Extended Data Fig. 8d). Finally, a human QSP model was created to predict the nACT complex levels in the bone marrow compartment resulting from specific doses and schedules of ISB 2001 administration enabling FIH calculation.

This QSP model was validated utilizing teclistamab to allow comparison of prediction to clinical outcome (Fig. 8a)^14^. The in vitro and in vivo experiments comparing ISB 2001 and teclistamab (Figs. 3i and 7h and Extended Data Fig. 7d,e) have been used to calibrate the models. The goodness-of-fit plots of these newly adapted models showed very good prediction by in vitro (Fig. 8c) and in vivo PKPD models (Fig. 8d). This gave us calibrated nACT levels for antitumor activity for both TCEs that could be used for comparisons to clinical data; however, these calibrated nACT levels were very different depending upon which preclinical dataset was used.

To understand which data (in vitro or in vivo) has the best predictive value, we used the QSP model to calculate the teclistamab doses required to achieve nACT corresponding to efficacy in the in vitro and in vivo experiments. These were then compared to the clinically approved dose levels of teclistamab^14^. The QSP simulation based only on the in vitro data, showed nACT levels of teclistamab above the EC_90_ of tumor killing at a dose of approximately 0.7 µg kg^−1^. Using in vivo datasets single-dose simulations showed that teclistamab RP2D regimen of 60, 300 then 1,500 µg kg^−1^ s.c. (including priming) achieved approximately the predicted 13%, 40% and 73% efficacy (EC_13_, EC_40_ and EC_73_) in the xenograft model. The teclistamab QSP model predicted that based on trimer formation, higher doses than 1,500 µg kg^−1^ s.c. will not lead to higher nACT (Fig. 8e). Clinically teclistamab was tested at doses up to 3,000 µg kg^−1^ s.c. without reaching an maximum tolerated dose (MTD) but without improved efficacy over the selected phase II dose of 1,500 µg kg^−1^ s.c., suggesting the model correctly predicted TCE’s clinical activity^35,36^). In addition, the clinically reported minimal efficacious dose (38.4 µg kg^−1^ i.v.) was predicted to correspond to the EC_17_ of in vivo efficacy, again implying that the model consistently predicted teclistamab’s behavior in clinical studies.

Following this validation, predictions were made for robust efficacy of ISB 2001, suggesting that nACT levels would continue to rise until doses of 2,000 µg kg^−1^ (Fig. 8e), likely due to avidity binding of two TAAs. A repeated s.c. dose of 180 µg kg^−1^ of ISB 2001 is expected to maintain the nACT level corresponding to in vivo EC_90_ in the bone marrow of patients with MM (Fig. 8f). In contrast, a teclistamab dose of 200 µg kg^−1^ achieves only the EC_50_ of killing corresponding to a 45 µg kg^−1^ dose of ISB 2001 (Fig. 8f). Thus, this model shows that ISB 2001 should achieve robust efficacy at doses lower than the teclistamab approved dose.

Finally, an MPAD simulation of nACT following a single dose^37^ was run for both ISB 2001 and teclistamab (Fig. 8g). Single doses with nACT maxima reaching the EC_20_, EC_30_ and EC_50_ were calculated. Using the most conservative EC_20_ calculation, teclistamab doses of 110 µg kg^−1^ s.c. were predicted. However, this low dose was in the range where teclistamab was delivered IV. Based on clinical results, the minimally efficacious dose of teclistamab was 38.4 µg kg^−1^ i.v.^36^. At that dose, the model predicted peak nACT levels in the bone marrow of a patient with MM equivalent to approximately the EC_17_ in the in vivo mouse KMS-12-BM xenograft model. Thus, we calculated an additional EC_17_ value for ISB 2001 and predicted that an s.c. dose of 10.5 μg kg^−1^, the peak nACT level in the bone marrow, will be equivalent to EC_17_; hence this represents the MPAD for ISB 2001 (Fig. 8g).

Additional evaluation of the starting dose was conducted by considering in vitro cytokine release assay results using ISB 2001 and teclistamab to determine a dose that should result in minimal cytokine release. The EC_30_ of the most sensitive in vitro cytokine release assay (TNF) was selected, and the QSP modeling suggested that this concentration would be achieved at a dose of 5 µg kg^−1^ (ref. ^38^). The similarity between the MPAD and the minimal in vitro cytokine release doses (10.5 μg kg^−1^ and 5 μg kg^−1^, respectively) strengthened the prediction of starting dose, and the lower of the two was selected for the FIH clinical studies of ISB 2001.

## Discussion

Outcomes for patients with r/r MM have substantially improved with the introduction of BCMA-targeted TCEs and CAR-Ts. Teclistamab showed similar efficacy to ide-cel, an anti-BCMA CAR-T cell, approved with median overall survival of 18.3 months^5^ and 19.4 months, respectively^4^. Recently, elranatamab has also been approved based on phase 2 MagnetisMM-3 study, with an overall response rate of 60% (ref. ^39^). Despite these encouraging results, patients continue to relapse. Therefore, it is imperative to develop new therapies to further extend patients’ remission.

Several mechanisms are potentially responsible for long-term remission^40^. Among many factors, depth of response (DOR) consistently correlates with improved overall survival and progression-free survival in patients with MM^41,42^. Patients with great DOR remained longer in remission than minimal residual disease (MRD)-positive patients^4,43,44^. Roughly 26.7% of patients treated with teclistamab were found MRD negative^5^. In addition, a large meta-analysis established the role of MRD negativity in long-term survival outcomes in patients with MM, indicating that potent and rapid killing of tumor cells required for MRD negativity is warranted for the success of MM therapy^41^. The dual targeting trispecific TCE, ISB 2001, showed superior potency compared to teclistamab and other tested TCEs, on cells with variable expression levels of CD38 and BCMA, mimicking the natural expression heterogeneity observed in patients with MM. Current clinical trials in patients with r/r MM are evaluating the combination of teclistamab with daratamumab^26^, an alternative strategy also targeting both CD38 and BCMA. ISB 2001 showed superior potency in vitro and in vivo relative to this combination, suggesting that dual TAA targeting by a single molecule, which possesses higher avidity against heterogeneous targets, is superior to combinations of approved therapeutics targeting BCMA and CD38. Of note, assessment of the CD38^+^ undifferentiated hematopoietic progenitors in CD34 humanized mice shows that neither ISB 2001 nor teclistamab treatment impact their numbers. Taken together this suggests that ISB 2001 could lead to superior MRD negativity associated with prolonged patient benefit relative to TCEs targeting a single TAA or two antibodies targeting the same receptors but having a mixed mode of action, a TCE and a monoclonal antibody in this case.

Another factor associated with low response durability is TAA downregulation^19,45,46^. This was first observed in the context of treatment with daratumumab^46–48^, where downregulation of CD38 was observed up to 6 months after therapy and this was associated with relapse. BCMA expression after T cell-mediated therapies is still under investigation, but antigen downregulation was observed with anti-BCMA CAR-T treatment^19,45^.

One mechanism of antigen downregulation is reversible antigen loss, mostly due to heterogeneity of target expression that enables expansion of pre-existing minor myeloma cell populations with lower expression of BCMA and/or CD38 (refs. ^19,49,50^). To address this, multi-targeted immunotherapies are being explored for the treatment of patients with MM with antigen downregulation or loss^45^. A BCMA/CD38-targeted bispecific CAR-T was described to have robust cytotoxicity against MM cells expressing either BCMA or CD38 (ref. ^51^). Similarly, the dual targeting by ISB 2001 induces strong antitumor responses, which could counteract antigen downregulation/loss of either CD38 or BCMA. When compared to teclistamab, ISB 2001 demonstrated a much higher cytotoxic potency in the context of BCMA^low^CD38^low^-expressing cells (KMS-12-BM) as well as in CD38 KO cells while slightly higher when compared in BCMA KO cells.

Antigen shedding could also interfere with binding of TCE^52^. Strong potency reduction was observed in all BCMA-targeted TCEs when incubated in the presence of sBCMA. ISB 2001 has a much lower sensitivity to soluble factors than monotargeting TCEs. This may contribute to DOR and prolonged antitumor response.

Despite being a rare mechanism, irreversible antigen loss has also been described^4,53^. Shortly after treatment initiation with anti-BCMA CAR-T, some MM cells may become BCMA^−/−^ and patients develop resistance. Such clones derive from MM cells, which have previously accumulated a heterozygous mutation on the *TNFRSF17(BCMA)* gene^54,55^. Heterozygous deletions were found in several MM-associated genes varying from 15% of *GPRC5D* to 4% of *TNFRSF17* (ref. ^54^). A recent report on a larger cohort, showed mono-allelic loss of *TNFRSF17* in 8.58% of newly diagnosed patients, associated with increased deletion frequency in other chromosomes^46^. The authors suggested that as more patients with MM receive monotargeted TCEs, an increasing number of patients may develop irreversible antigen loss and treatment with dual-antigen-targeting therapies, such as ISB 2001, could prevent such escape by killing cells expressing only BCMA or CD38, even if at a lower potency.

T cell quality also plays a role in the primary response and in long-term treatment outcome. T cell exhaustion in patients with MM has been correlated with progressive disease^56^. When assessing cytotoxicity mediated by patients’ T cells, ISB 2001 showed a potency 100-fold higher than teclistamab. Of note, in one ex vivo sample from a patient with MM who relapsed after elranatamab treatment, ISB 2001 induced superior cytotoxicity compared to teclistamab, highlighting the high cytotoxic potency of ISB 2001, which is capable of overcoming escape mechanisms linked to monotargeted TCEs.

The most common adverse event observed during the treatment with TCEs is cytokine release syndrome (CRS). Patients with severe or life-threatening CRS require intensive care and safety mitigation measures are warranted^57,58^. Despite exhibiting 10–100-fold higher cytotoxic potency than teclistamab, ISB 2001 induced a similar cytokine secretion in vitro. These data are in line with previous observations^59^ indicating that the threshold for T cell activation and cytolytic activity requires 10,000-fold lower concentration of TCR stimulation than for the onset of CRS. In addition, assessment of the on-target/off-tumor activity of ISB 2001, which could contribute to CRS, showed a profile more similar to BCMA-targeted TCEs rather than a CD38-targeted TCE. These data provided biological evidence that trispecific TCEs can achieve high cytotoxic potency without a corresponding increase in cytokine release.

To define ISB 2001 FIH dose, a QSP modeling approach was used with teclistamab as benchmark, leading to several advantages. First, TCEs can be optimized entirely for activity against human tumors without compromising preservation of cross-reactivity to animal species. Second, by increasing the starting dose using QSP modeling, fewer patients will be exposed to subtherapeutic doses in the FIH trial. The proposed 5 µg kg^−1^ starting dose is 50–100-fold higher than the MABEL-based starting dose (ranging from 0.045 μg kg^−1^ to 0.1 μg kg^−1^). Third, such models could minimize the use of cynomolgus monkeys supporting the 3Rs principles even when evaluating fully cross-reactive molecules.

In conclusion, ISB 2001 is a trispecific TCE with the potential to induce deep responses in patients with r/r MM, by overcoming several factors that limit the response to other BCMA- and CD38-monotargeted TCEs. Based on the preclinical data, a phase I clinical trial of ISB 2001 in patients with r/r MM has been initiated using the FIH dose calculated employing the QSP model (NCT05862012).

## Methods

Our research complies with all relevant ethical regulations. Refer to each specific section for details in the committee and institution that approved the study protocol.

### Production of ISB 2001 and other antibody constructs

ISB 2001 and other antibody constructs were expressed transiently in CHO-S cells (cGMP banked, Invitrogen, cat. no. A1136401). Typically, cells were prepared at 8 million cells per ml in CD-CHO medium (Gibco). Cells were then co-transfected with engineered chains vectors and a vector encoding Epstein–Barr virus nuclear antigen‐1 (EBNA‐1) using polyethyleneimine (PEI) at 37 °C. Four hours post-transfection, the cell culture was diluted 1:1 in PowerCHOTM 2 (Lonza) supplemented with 4 mM l-glutamine and incubated for 10 days with orbital shaking at 32 °C, 5% CO_2_ and 80% humidity. Clarified cell culture supernatants containing the recombinant proteins were prepared by centrifugation followed by filtration. Antibodies were purified by protein A^10^, followed by a second step of purification by cation exchange chromatography to reach monodispersity >95%, as judged by analytical SE-HPLC. ISB 2001 could be made available upon agreement.

Expression vectors for teclistamab and alnuctamab were synthesized using publicly available sequences information. Molecules were produced transiently in CHO-S cells. Teclistamab was expressed as two separate monoclonal antibodies with anti-BCMA or anti-CD3 binding domains and further reconstituted^60^. For the preparation of the 2 + 1 CrossFab IgG alnuctamab, cells were transfected with the four corresponding expression vectors, using an optimal expression vector ratio^6^. Antibodies were purified as above and then transferred to an appropriate buffer.

### Production of EM801 (83A10-TCBcv) in stable HD-BIOP3 pools

EM801 was deemed to be 83A10-TCBcv, described in WO2018083204 as per the data comparison between WO2018083204 and a published article^6^. Sequences of 83A10-TCBcv were extracted from WO2018083204. In brief, HD-BIOP3 cells were electroporated (Neon electroporation system, Thermo Fisher) with the ATUM Leap-In transposase mRNA and a donor vector containing the genes for the expression of the two light chains and two heavy chains of 83A10-TCBcv. These genes, as well as the glutamine synthetase gene (selection marker), were flanked by two inverted terminal repeats for transposase-driven integration in the host cell’s genome. Selection was performed by transferring cells in a glutamine-free medium and carried out until cell recovery (viability >90% and 20–25-h doubling time). After selection, a 12-day fed-batch production was performed in a shake flask (37 °C, 5% CO_2_, 80% humidity and 150 rpm), with a temperature shift to 32 °C on day 4. At the end of the process, clarified cell culture supernatants containing the recombinant proteins were prepared by centrifugation followed by filtration. EM801 was purified as described in the previous section.

### Biophysical characterization of antibodies

SE-HPLC was run on a TSKgel G3000SWXL 7.8 mm × 30 cm L column with 5-μm particles and 250 Å pores (Tosoh Bioscience) at room temperature with 0.1 M sodium phosphate buffer, 0.15 M sodium chloride, pH 6.8 as eluent at 1 ml min^−1^ flow rate on an HPLC Alliance 2695 (Waters) or an Acquity Arc HPLC (Waters) with column heater and either UV/Vis detector (2487 or 2489 from Waters) or PDA detector (2996 or 2998 from Waters). Capillary gel electrophoresis was performed on a Beckman Coulter PA 800 system with DAD/PDA detector (Diode Array Detector/Photodiode Array Detector). The IgG Purity kit (Beckman Coulter) was used according to the manufacturer’s instructions. Samples were desalted before the run and mixed with iodoacetamide (Sigma-Aldrich) for nonreduced samples or with β-mercaptoethanol (Sigma-Aldrich) for reduced samples. Resulting electropherograms were analyzed and integrated using the Empower software (Waters). Calorimetric measurements were carried out on a VP-DSC differential scanning calorimeter or a MicroCal PEAQ-DSC differential scanning calorimeter (Malvern Instruments) using a 1 °C min^−1^ heating rate. ISB 2001 was used at a concentration of 1–2 mg ml^−1^ in PBS. The molar heat capacity of the molecule was estimated by comparison between duplicate samples containing identical buffer, from one of which the protein had been omitted. The partial molar heat capacities and melting curves were analyzed using standard procedures (non-two-state model) in the manufacturer software.

### BCMA–APRIL and BCMA–BAFF blockade assay

Blockade of the BCMA–APRIL or BCMA–BAFF interaction upon binding of anti-BCMA Fab to BCMA was assessed by SPR on a Biacore 8K+ instrument (Cytiva). Human APRIL HisTag protein (Acrobiosystems, APL-H5244) or human BAFF HisTag protein (Acrobiosystems, BAF-H5248) was immobilized on an anti-histidine-coated sensor chip (Cytiva). Pre-mixed solutions of 50 nM human BCMA FcTag protein (Acrobiosystems, BC7-H5254) and of anti-BCMA Fab at various concentrations (0, 25, 50 and 200 nM), were individually flushed over immobilized APRIL or BAFF.

### Competition assays by BLI

Competition of Fab domains or antibody constructs was assessed using BLI. Measurements were carried out on an OctetRED96e instrument (Sartorius). Streptavidin biosensors (Sartorius) coated with biotinylated human CD38 protein (Acrobiosystems, CD8-H82E7) were dipped into a solution of a saturating Fab or antibody, followed by a successive dip into a mixed solution of the same and of competing Fab or antibody. The putative binding site of ISB 2001 was mapped to the surface of human CD38 based on competition profile to daratumumab and isatuximab using their respective published structures (Protein Data Bank (PDB) 7DHA and 4CMH).

### Affinity measurements by SPR

SPR measurements were performed on a Biacore 8K+ instrument (Cytiva).

For binding to CD3εδ, BCMA and CD38, biotinylated human CD3ε&CD3δ protein (Creative Biomart, CD3E & CD3D-377H), biotinylated human CD38 protein (Acrobiosystems, CD8-H82E7) or biotinylated human BCMA protein (Acrobiosystems, BCA-H82E4) were immobilized on a Biotin Capture (CAP) sensor chip (Cytiva) and increasing concentrations of ISB 2001 were flushed onto the immobilized ligand. Concentrations ranged from 1 µM to 1.37 nM or from 100 nM to 0.05 nM in a 1:3 dilution series for binding to CD3εδ and BCMA or to CD38, respectively. Data were fitted to a steady-state affinity model (CD3εδ) or to a 1:1 kinetic model (BCMA and CD38). The same procedure was followed for the assessment of the binding of alnuctamab, teclistamab and EM801 to CD3εδ and BCMA, using concentrations from 400 nM to 0.55 nM to CD3εδ and from 50 nM to 0.07 nM (alnuctamab and teclistamab) or from 100 nM to 0.14 nM (EM801) to BCMA in 1:3 dilution series.

For binding to the Fcγ receptors, the antibody constructs were immobilized on a Protein G sensor chip (Cytiva) for human FcγRIA, FcγRIIA, FcγRIIB and FcγRIIIA binding or on a Protein L sensor chip (Cytiva) for FcRn binding and increasing concentrations of the receptors were flushed onto the immobilized antibodies. Measurements to FcRn were conducted at pH 6.0, whereas measurements to the other Fcγ receptors were performed at neutral pH. Binding to FcγRIA was fitted using a 1:1 kinetic model and binding to FcγRIIA, FcγRIIB, FcγRIIIA and FcRn were fitted using a steady-state affinity model.

### Human T cell activation

Anti-human CD3 produced as human IgG1 LALA were coated at increasing concentrations up to 200 nM in PBS in a 96-well plate overnight (ON) at 4 °C. Isolated T cells from buffy coats (EasySep Human T Cell Isolation kit, STEMCELL technologies, 17951) were added to the coated plate at 10^6^ cells per ml and incubated at 37 °C for 48 h. T cell activation was measured as the proportion of live CD8^+^ T cells expressing CD69 by flow cytometry (Cytoflex-S, Beckman Coulter).

### Human primary samples and cell lines

BMAs or peripheral blood samples from patients with MM without sex distinction were obtained from University Hospital Geneva (Geneva ethical committee 2021-02416), Oxford University Hospitals (Oxford Clinical Research Ethics Committee (17/SC/0572) and the HaemBiobank Governance Committee (BBProj-27.0 and BBProj-13.0)) and Nantes Université (MYRACLE cohort^61^; NCT03807128) with written informed consent under each site ethical approvals. Human PBMCs (hPBMCs) from HDs and patients with MM and BMMCs were isolated using Ficoll gradients. KMS-12-BM (DSMZ, cat. no. ACC551), MOLP-8 (DSMZ, cat. no. ACC569), NCI-H929 (Sigma-Aldrich, cat. no. 9505041), NCI-H929 deficient for BCMA or CD38 (Methods), were tested as *Mycoplasma*-free and cultured in the medium recommended by the supplier in a humidified atmosphere of 5% CO_2_ at 37 °C. The identity of the cell lines was confirmed at early passages and at the end of the culture using short tandem repeats performed according to Microsynth guidelines. Human samples were used in their totality.

### Generation of NCI-H929 BCMA KO and NCI-H929 CD38 KO cells

NCI-H929 BCMA KO and CD38 KO cell lines were derived from original NCI-H929 cells (Sigma-Aldrich, cat. no. 9505041) by targeting the first exon of the *TNFRSF17(BCMA)* or *CD38* gene using clustered regularly interspaced short palindromic repeats (CRISPR)/Cas9 technology (guide RNA target sequences CAATAACGCTGACATGTTAG and TACTGACGCCAAGACAGAGT, respectively). The NCI-H929 cell line was transfected using 4D-nucleofector (Lonza) according to the manufacturer’s recommendation, then sorted using Melody sorter (BD Biosciences) to generate cell pools. The lack of BCMA or CD38 expression was further verified by flow cytometry using QIFIKIT reagent (Agilent DAKO, K0078) and mapping breakpoint analysis using TA-cloning, Sanger sequencing and ICE (Synthego) software.

### Redirected lysis assay

Cell lines were labeled with 0.5–1 µM eFluor 670 dye (Invitrogen, 65-0840-85) and co-cultured for 72 h with hPBMCs or for 48 h with isolated T cells from HD- or patient sample (PS)-PBMCs or BMA at an effector to target ratio (E:T) of 5:1 with increasing concentration of the tested molecules diluted in RDL medium (Supplementary Table 2). In assays evaluating the effect of soluble factors on the cytotoxic potency, soluble BCMA (150 ng ml^−1^), APRIL (100 ng ml^−1^) and soluble CD38 (2.8 ng ml^−1^) were added to the cultures, alone or in combination. When indicated, effector cells were labeled with 5 µM of eFluor450 dye (Invitrogen, 65-0842-85). Tumor cell killing was measured as the percentage of dead target cells or as the decrease of live target cells count normalized to the untreated condition. Data acquisition was performed by flow cytometry (IntelliCyt iQueScreenerPlus, Sartorius) and analysis was performed using ForeCyt Software (Sartorius). T cell activation was measured as the percentage of live CD4^+^ or CD8^+^ T cells expressing CD25 and the loss of eFluor450 dye was used to measure T cell proliferation (see Supplementary Table 3 for antibody references).

### High-density PBMC assay

PBMCs from HDs were cultured at 10^7^ cells per ml for 48 h, then incubated for an additional 48 h at 0.5 × 10^6^ cells per ml with increasing concentrations of tested molecules in HD-PBMC medium (Supplementary Table 2). T cell activation was measured as the percentage of CD4^+^ or CD8^+^ T cells expressing CD25, granzyme B and perforin using Cytoflex-S cytometer. Alternatively, quantification of the events of CD4, CD8, CD14, CD20 and CD56 was assessed. Analysis was performed using CytExpert software (Beckman Coulter).

### Confocal live imaging

KMS-12-BM cells were stained with 15 μM of CellTracker Blue Dye (Invitrogen, C2111) and plated with T cells from HDs labeled with 5 μM CellTracker Orange Dye (Invitrogen, C2927) at an E:T of 5:1. Tested antibodies were labeled using Zenon Alexa Fluor 647 Goat IgG Labeling kit (Invitrogen, Z25608). Labeled molecules (10 nM) were added to the cells on ibiTreat pre-coated slides (Ibidi) for 4 h at 37 °C. Live microscopy was carried out using a Zeiss LSM 800 inverted confocal microscope incubation system (Carl Zeiss). Images were processed with Imaris software (Oxford Instruments).

### Tumor T cell interaction by live imaging

KMS-12-BM cells were labeled with 2 μM CellTracker Red Dye (Invitrogen, C34552) and co-cultured for up to 24 h with isolated T cells, labeled with 2.5 μM CellTracker Green Dye (Invitrogen, C2925) at an E:T ratio of 5:1 with tested samples at doses ranging from 2000-2 pM. Images were acquired on Incucyte S3 with ×20 objective every 30 min for 6 h and every hour up to 24 h.

### Cytokine release quantification

Cytokine release in the culture supernatant of RDL and HD-PBMC assays were assessed by LEGENDplex Multi-Analyte Flow Assay kit (BioLegend) according to the manufacturer’s instructions. Human CD8/NK Mix and Matched Subpanel was used to quantify IFNγ, TNF, granzyme B, perforin, IL-2, IL-6 and IL-10. Samples were acquired on a Cytoflex-S cytometer and data were analyzed with LEGENDplex online tool. Lower limits of detection (LLOD) were set for each cytokine using the lowest concentration of the calibration curve or quality control sample with a coefficient of variation below 30 %. Upper limits of detection (ULOD) were set for each cytokine using the highest concentration of the calibration curve. When cytokine release was below the LLOD or above the ULOD the value of the sample was set at LLOD or ULOD respectively.

Cytokine release in serum and tumor supernatant samples (undiluted) was assessed by multiplex Luminex quantification, according to the manufacturer’s instructions. The cytokine and chemokine 34-Plex Human ProcartaPlex Panel 1A kit (Invitrogen, EPXR340-12167-901) was used. Acquisition was carried out with a Luminex 200 instrument and data were analyzed with ProcartaPlex Analyst 1.0 software. Cytokine concentration was normalized to the upper and lower limit of quantification (defined using ProcartaPlex standards) for each cytokine/chemokine. The final concentration was then normalized per gram of tumor.

### Multiple mode of action killing assay

Human PBMCs labeled with 5 µM eFluor450 dye (Invitrogen, 65-0842-85) were co-cultured with KMS-12-BM cell line previously labeled with 1 µM eFluor 670 dye (Invitrogen, 65-0840-85) in MMoAK medium (Supplementary Table 2) at an E:T of 5:1. Effector and target cells were incubated with increasing concentrations of ISB 2001 or teclistamab. Daratumumab (Darzalex, Janssen Biotech) was tested at 100 nM either alone or in combination with ISB 2001 or teclistamab. After 48 h, MM cells were stained for viability (Live/Dead Green) and tumor cell killing was measured as the percentage of dead target cells using an IntelliCyt iQueScreenerPlus flow cytometer (Sartorius). Analyses were performed using ForeCyt Software (Sartorius).

### Specific antibody-binding capacity by flow cytometry

Specific antibody-binding capacity of human CD38 and human BCMA was measured using QIFIKIT (Agilent DAKO, K0078) or Human IgG Calibrator (BioCytex, CP010) according to the manufacturer’s instructions. Mouse anti-human CD38, mouse anti-human BCMA and mouse isotype IgG1 were used as primary antibodies at saturating concentration with QIFIKIT. Daratumumab (Darzalex, Janssen Biotech) and anti-human BCMA (produced in-house from vectors synthesized using publicly available sequence information) were used as primary antibodies at saturating concentration with Human IgG Calibrator kit.

### Cell-based affinity assay

KMS-12-BM, MOLP-8, NCI-H929, NCI-H929 deficient for BCMA or CD38 or purified T cells from hPBMCs were incubated with increasing doses of tested molecules in a 96-well plate at 4 °C in the dark for 30 min, washed with FACS buffer supplemented with 0.05% sodium azide, then stained with Live/Dead NIR. Binding was detected using an APC-labeled anti-human Fc monoclonal for MM cells or a PE-labeled anti-human Fc monoclonal for T cell secondary antibody. For binding to T cells, additional staining with anti-CD38 FITC antibody was performed. Acquisition was performed on an IntelliCyt iQueScreenerPlus flow cytometer (Sartorius). The geometric mean of fluorescence intensities (MFI) of the viable single cells (for cell lines) or viable CD38^−^ T cells (for T cells) was extracted using ForeCyt Software (Sartorius). The values of MFI from the control antibody were subtracted to the matching concentration MFI of the tested antibodies to generate the relative fluorescence intensity.

### Ex vivo cytotoxic assay on samples from patients with MM

BMMCs or peripheral blood of patients were co-cultured at 1–2 × 10^6^ cells per ml with increasing concentration of tested molecules in PS medium for 17–32 h at 37 °C (Supplementary Table 2). Samples were acquired using Cytoflex-LX cytometer (Beckman Coulter) or LSRFortessa cytometer (BD Biosciences). Tumor cell killing was calculated as the decrease of the remaining live target cell counts CD138^+^ after treatment and normalized to the untreated condition. T cell activation was measured as the percentage of CD8^+^ T cells expressing CD69.

### Mice

NCG (NOD-Prkdcem26Cd52Il2rgem26Cd22/NjuCrl) mice were purchased from GemPharmatech Co. and used in PK evaluation at Crown Bioscience and in a subcutaneous tumor model at Crown Bioscience in accordance with reviewed and approved Institutional Animal Care and Use Committee protocols. hFcRn Tg32 SCID mice (B6.Cg-Fcgrttm1Dcr Prkdcscid Tg(FCGRT)32Dcr/DcrJ; JAX stock no. 018441) were purchased from The Jackson Laboratory and used directly at The Jackson Laboratory for PK evaluation in accordance with JAX Institutional Animal Care and Use Committee protocols. NSG ((NOD.Cg-Prkdcscid Il2rgtm1Wjl/SzJ) and HIS-NXG (human immunized system-NOD-Prkdcscid-IL2rgTm1/Rj, reconstituted with human cord blood CD34^+^ cells) mice were purchased from Janvier France and used at the animal facilities of the University of Lausanne in accordance with protocols approved by the veterinary authorities of the Canton de Vaud. All mice were maintained under standardized environmental conditions in rodent cages (20–26 °C temperature, 40–70% relative humidity and 12-h light–dark cycle). Mice received irradiated food and bedding and 0.22-µm-filtered drinking water ad libitum. Tumor-bearing mice were killed when the tumor volume reached >1,000 mm^3^ in accordance with approved protocols.

For PK experiments, mice of either sex were used, based on availability and bodyweight (to ensure ethical blood sampling as maximum blood volume is determined by weight). Only female mice were used in studies with tumors to respect the need for social housing and the 3Rs after randomization based on tumor volume. Mice with the same treatment were co-housed to minimize the animal stress and the risk of experimental error.

### PK evaluation in NCG and hFcRn mice

The PK profile of ISB 2001 was assessed in NCG mice following either i.v. or s.c. administration at 0.5 mg kg^−1^ or 5 mg kg^−1^ on day 0. For the study comparing ISB 2001 and teclistamab, mice also received 200 mg kg^−1^ of IgG (Boxin Biotech) i.v. on day −1, day 6 and day 13, to mimic the conditions in the improved KMS-12-BM xenografted model (see below). Micro-samplings (25–30 µl blood) were collected at 15 min, 4 h, 1 day, 2 days, 4 days, 7 days, 9 days, 15 days and 21 days post-dose. Plasma concentrations of ISB 2001 and teclistamab were determined using an electrochemiluminescence (ECL) method using a Meso Scale Discovery (MSD) platform. For PK evaluation in hFcRn Tg32 SCID mice, micro-samplings were collected at 5 min, 1 day, 3 days, 7 days, 10 days, 14 days, 17 days, 21 days, 24 days and 28 days after i.v. administration of ISB 2001 (5 mg kg^−1^). Plasma concentrations of ISB 2001 were assessed using Mabtech (ref. 3850-1AD-6) total human IgG Fc ELISA kit. All PK calculations were performed using noncompartmental analysis with Phoenix WinNonlin v.8.3 (Certara).

### ECL quantification using MSD from mouse plasma

Antibodies in mouse plasma were quantified by an exploratory ECL-based immunoassay method developed using the MSD platform. Assays were performed as per the manufacturer’s instructions, using their reagents except for plate coating, which was carried out overnight at 2–8 °C with recombinant human BCMA HisTag protein at 2.0 µg ml^−1^ (ISB 2001) or 1.0 µg ml^−1^ (teclistamab), and for detection, where sulfo-tag conjugated anti-idiotypic antibody of CD38 domains of ISB 2001 at 2 µg ml^−1^ or a mixture of biotin conjugated anti-idiotypic antibody of CD3 domains of teclistamab at 1 µg ml^−1^ and 0.25 µg ml^−1^ streptavidin sulfo-tag was used. Casein was used in the blocking step and diluent buffer.

### Tumor models

The 6–7-week-old NSG or NCG female mice were engrafted s.c. with 1 × 10^7^ KMS-12-BM or 1 × 10^7^ NCI-H929 tumor cells, respectively and inoculated intraperitoneally (i.p.) with 1 × 10^7^ PBMCs from human HDs on the same day. When tumors reached an average of 150 mm^3^, mice were randomized based on the tumor volume and injected i.v. on the following day with vehicle, ISB 2001 or CD3DU control at the indicated doses once per week for 3 weeks. Tumors and spleens of satellite animals (that received a single dose of molecules) were collected at day 2 and 6 post-dose. Single-cell suspensions were analyzed by flow cytometry. Human cytokine detection was performed with a Luminex assay on the serum and tumor supernatant. When comparing ISB 2001 and teclistamab, mice were injected i.v. with 200 mg kg^−1^ IVIG (Privigen) 1 day before treatment injection, a pretreatment mimicking physiological levels of irrelevant immunoglobulin to compensate for the mouse B cell deficiency.

HIS-NXG mice (24–30-week-old mice reconstituted with CD34^+^ from five donors) were stratified into control and treatment groups based on tumor size and donors (Extended Data Fig. 6f). At 24 h after pre-conditioning with IVIG (i.p.), mice were treated s.c. with ISB 2001, teclistamab, daratumumab or both teclistamab and daratumumab.

Tumor volumes were measured by caliper and calculated using the formula: *V* = (*L* × *W* × *W*)/2, where *V* is the tumor volume (mm^3^), *L* is the longest tumor dimension and *W* is the longest tumor dimension perpendicular to *L*. The last observation carried forward was applied to display the tumor volume. An exclusion criterion was that if animals demonstrated signs of graft-versus-host disease, a common effect in systemic PBMCs of humanized mice, they were killed before the study end point and excluded from the analysis.

### Assessing progenitor cells in humanized mice bone marrow

The 30–34-week-old HIS-NXG mice were pretreated with 200 mg kg^−1^ IVIG (i.p.) 24 h before s.c. injection with 1.5 mg kg^−1^ ISB 2001, teclistamab, CD38 × CD3 control antibody or with PBS. CD34^+^ humanized mice were reconstituted from three different donors, equally distributed in the treated groups. Three days after treatment with the different molecules, mice were killed and both femurs were collected. CD38 expression on progenitor cells in the bone marrow was analyzed by flow cytometry.

### Sample preparation and staining for flow cytometry

To obtain the cell suspension, femurs were flushed with PBS, spleens were mashed through a 70-μM nylon cell strainer and tumors were dissociated using a tumor dissociation kit from Miltenyi (130-095-929) in a gentleMACS dissociator. Cell suspensions were incubated with viability dye, human and mouse Fc Block for 15 min at 4 °C in FACS buffer (PBS and 2% FBS), followed by surface staining with an antibody cocktail (or relative controls) for 30 min at 4 °C in FACS buffer. Samples were acquired on the Cytek Aurora instrument and analyzed with FlowJo v.10.8.1.

### QSP model

The QSP model was built by combining a minimal PBPK model with a target-binding model based on the published method^27,33^. The method of Betts was modified to include binding interactions of three binders (CD3, CD38 and BCMA) and the available preclinical datasets. The building, benchmarking and prediction strategy of the QSP model are described here sequentially (Fig. 8a).

#### Model building

The QSP model is constituted by sequentially generated mathematical models. Each model was built by an iterative process using assumptions and experimental data to define key parameters applied in subsequent models. MATLAB/Simbiology v.R2021a (The MathWorks), was used for all PKPD/QSP analyses and simulations.

#### Target-binding model

This model, based on equilibrium binding kinetics (Fig. 8b), was created using SPR binding data, cell numbers along with CD38 and BCMA receptor densities on MM cell lines, CD3 receptor density on T cells^27^ and internalization *t*_1/2_ for each target from the literature^62–64^. This model assumes that the following complexes could be formed: (1) dimers of ISB 2001 with either CD3, CD38 or BCMA; (2) trimers of ISB 2001-CD3 with one of the targets (CD38 or BCMA) or with ISB 2001 with CD38 and BCMA on the tumor; and (3) tetramers of ISB 2001-CD3 with both targets on the tumor (CD38 and BCMA). The sum of trimers and tetramers consisting of TCE, CD3, BCMA and/or CD38 were assumed to be equipotent pharmacologically active species (ACT) that drive T cell activation and tumor cell killing. A single compartment was used for the binding model consistent with the in vitro test conditions used for RDL (Extended Data Fig. 8a). To facilitate the translation across various experimental conditions in vitro, in vivo and ultimately to clinical scenario, ACT was expressed as normalized ACT per tumor cell (nACT).

#### In vitro cytotoxicity and T cell activation model

**Extended Data Fig. 9:**
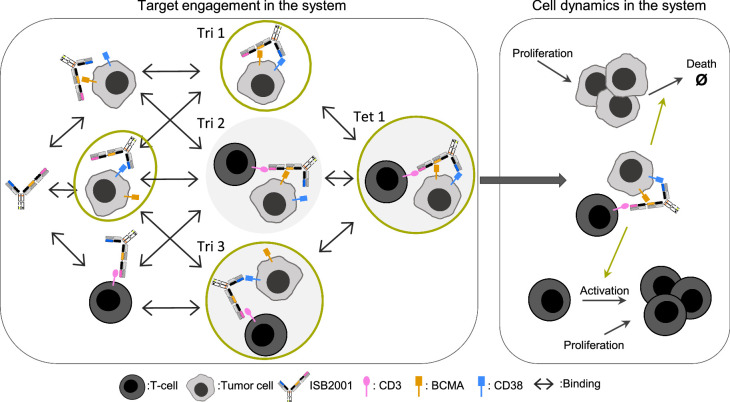
QSP schematics. In vitro killing model with target engagement on left and cell dynamics on right. Species not produced by teclistamab due to lack of a CD38 binder are circled in green and trimers labeled Tri 1, Tri 2, Tri 3 and tetramer Tet 1. Active species (Tri 2, Tri 3, Tet 1) are shown with a light gray background.

This model simulates tumor cell growth and degradation, as well as T cell proliferation and activation consistent with an RDL assay at 72 h. The tumor cytotoxic effect of ISB 2001 was modeled through the formation of nACT, which stimulated the tumor cell degradation rate as a sigmoidal function of the nACT. T cell activation and proliferation was modeled as a sigmoidal function of ACT per T cell, stimulating the activation rate and proliferation rate, respectively (Extended Data Fig. 9).

#### In vivo mouse PKPD model

The PK data from NCG, NSG and hFcRn TG32 SCID mice were fitted to a minimal PBPK model^33^. This model assumed that ISB 2001 behaves like a typical IgG antibody without cross-reactivity of its binding domains to mouse. This model gave a dose–time–exposure relationship, which was used in mouse PKPD modeling. The model estimated clearance (CL) from single-dose PK data of hFcRn TG32 SCID was scaled to a human model (Extended Data Fig. 8d).

**Extended Data Fig. 10:**
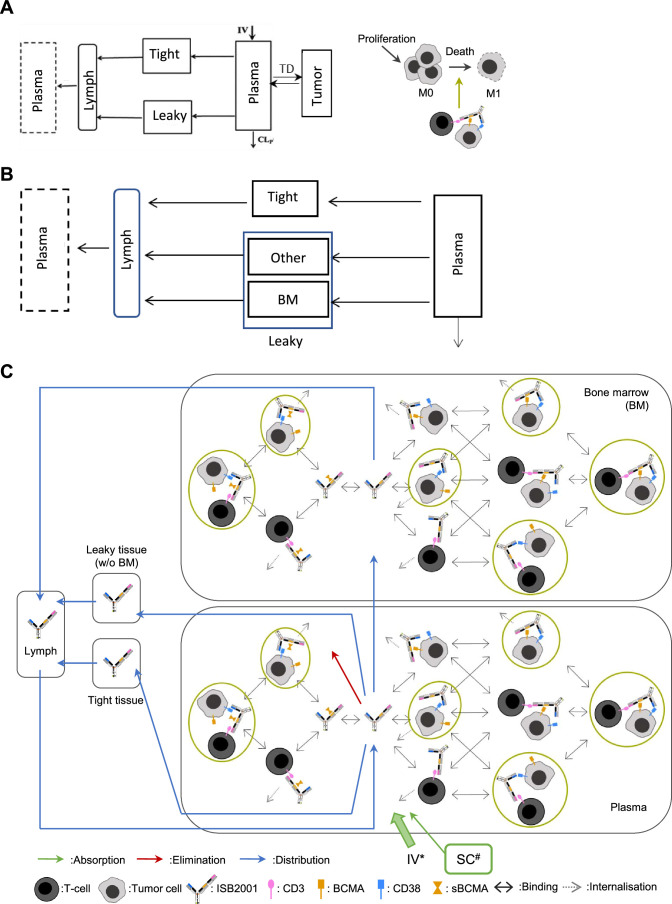
QSP Schematics. **(a)** In vivo mouse PK/PD model with PK model (left) and tumor compartment cell dynamics upon trimer formation (right). **(b)** Human PBPK model with specific bone marrow compartment (illustrated in blue box). **(c)** Human QSP model with binding dynamics in compartments. Species in green circles are not produced by teclistamab due to lack of CD38 binder.

To develop a mouse PKPD model^27^, an additional tumor compartment was added to the PBPK model developed earlier^33^, to estimate the nACT profile in the tumor. It was assumed that following i.v. administration of PBMCs, T cells were able to distribute to the tumor and back to the central compartment with fixed rate constants; the CD3 density on T cells, permeability and diffusivity of TCE into the tumor were obtained from the literature^27^. As PD data were available from early post-ISB 2001 administration, but limited later in the treatment due to tumor regression, trafficking rather than proliferation was assumed to be the dominant mechanism of delivering T cells to the tumor in this model. Tumor regression was described as a sigmoid function of the nACT profile in the cell distribution transduction model explained by Betts et al.^27^ (Fig. 7 and Extended Data Fig. 10a).

#### Human QSP model

Finally, a human QSP model integrating the target engagement and the PBPK model was developed (Extended Data Fig. 10b). A published minimal PBPK model, including blood, leaky tissue, tight tissue and lymph was adapted to estimate the nACT profiles in the bone marrow^33^. The leaky tissue compartment in the base model was split into two subcompartments: bone marrow and leaky tissues^65,66^. For ISB 2001, all physiological parameters, except for CL, were fixed to the parameters published for typical IgG^67,68^. The clearance of ISB 2001 in the human model was estimated by allometric scaling of CL from hFcRn TG32 SCID mice using an exponent of 0.85 (ref. ^28^). To model subcutaneous injection bioavailability and absorption, rates were taken from the literature^69^. Additional assumptions were obtained from the literature: cell counts^70–78^ (https://my.clevelandclinic.org/), soluble BCMA and CD38 levels^2,23^, CD3, CD38 and BCMA expression levels^2,6,76^. This model predicted the nACT profile in the bone marrow over time following either i.v. or s.c. administration. This nACT profile was then related to efficacy based on the calculated nACT levels at a specific EC_x_ (tumor cell killing) from the in vitro and in vivo PD models. The final QSP model used outputs from all previous models either as parameters or linked active species levels to possible outcomes. (Extended Data Fig. 10c).

#### Calibration and benchmarking

The QSP models were recalibrated and fine-tuned to describe the ISB 2001 and teclistamab preclinical datasets. The teclistamab target-binding model was achieved by adapting the ISB 2001 target-binding model to use teclistamab-specific affinity parameters for BCMA and CD3 binding determined by SPR and setting the CD38 affinity to ‘0’ (Extended Data Fig. 8a). A similar approach was taken to adapt the in vitro model (Extended Data Fig. 9). For the in vivo modeling, the same minimal PBPK model was used with single-dose PK data for teclistamab to determine the dose–exposure relationship (Extended Data Fig. 10a).

#### Human model

The teclistamab clinical PK data^36^ were used to build the human PBPK model^33^, including bone marrow, blood, leaky tissue, tight tissue and lymph (Extended Data Fig. 10b).

### Statistical analysis

Statistical analysis and graphs were generated using GraphPad Prism software (GraphPad Software). For in vitro experiments, no statistical methods were used to predetermine sample size, the experiments were not randomized and the Investigators were not blinded to allocation during experiments and outcome assessment. A nonlinear one-site binding (hyperbola) regression was applied to calculate *K*_d_ in cell binding assays. To allow *K*_d_ calculations, tested concentrations inducing a >20% hook-effect were excluded. The percentage of tumor cell killing, killing of CD138^+^ cells and T cell response (activation or proliferation) were fitted with four-parameter logistic nonlinear regression with a variable slope. EC_50_ values were excluded when the *R*^2^ of the fitting curve was <0.7, the observed maximum response was <25% or the calculated EC_50_ values were below or above the tested concentrations.

EC_50_ and *K*_d_ were log_10_-transformed before performing any statistical comparison. The normality of data was checked using the Shapiro–Wilk or Kolmogorov–Smirnov test and homogeneity of the variance was tested using a Bartlett or Spearman’s test. All EC_50_ and *K*_d_ statistical comparisons were performed two-sided. Differences between two groups were analyzed by a multiple paired *t*-test using Holm–Šidák’s or false discovery with the two-stage step-up (desired false discovery rate *Q* set at 1%) method. A classical one-way analysis of variance (ANOVA), repeated measures (RM) one-way ANOVA (assuming sphericity), two-way ANOVA or mixed-effects model (REML) were used for multiple group comparisons or using Friedman or Kruskal–Wallis tests for nonparametric comparisons. Post-hoc comparisons were performed for parametric testing using Tukey’s multiple comparisons between all groups, a Dunnett’s test for comparisons with a control group, the uncorrected Fisher’s LSD comparison or a Šidák’s multiple comparison for two samples in a specific group. For nonparametric post-hoc comparisons, Dunn’s multiple comparisons test was used. For in vivo studies, 7–9 mice per group were used based on power calculations using G*Power (90% power and 0.05 error probability)^77^. In tumor models, a randomization based on the tumor volume was carried out before starting the treatment. Data collection and analysis were performed blind for the outsourced in vivo experiments performed in Crown Bioscience and The Jackson Laboratories but not for the other models and experiments. *P* ≤ 0.05 was considered significant. The number of biological replicates, independent experiments performed and statistical analysis performed are stated in all figure legends.

RSE (relative standard error) was calculated in MATLAB at the EC_50_ of each predication shown in the modeling goodness-of-fit plots.

### Reporting summary

Further information on research design is available in the Nature Portfolio Reporting Summary linked to this article.

## Supplementary information

Supplementary Information Supplementary Figs. 1 and 2.

Reporting Summary

Supplementary Tables Supplementary Table 1: SPR-derived constant of association (*K*_on_), constant of dissociation (*K*_off_) and equilibrium dissociation constant (*K*_d_) of tested antibodies to human CD3εδ, human BCMA and human CD38. Supplementary Table 2: culture medium for in vitro and ex vivo assay. Supplementary Table 3: antibodies, clones, supplier, catalog number, dilution and assay where they were used.

## Source data

Source Data Fig. 1 Raw data used for Fig. 1.

Source Data Fig. 2 Raw data used for Fig. 2.

Source Data Fig. 3 Raw data used for Fig. 3.

Source Data Fig. 4 Raw data used for Fig. 4.

Source Data Fig. 5 Raw data used for Fig. 5.

Source Data Fig. 6 Raw data used for Fig. 6.

Source Data Fig. 7 Raw data used for Fig. 7.

Source Data Fig. 8 Raw data used for Fig. 8.

Source Data Extended Data Fig. 1 Raw data used for Extended Data 1.

Source Data Extended Data Fig. 2 Raw data used for Extended Data 2.

Source Data Extended Data Fig. 3 Raw data used for Extended Data 3.

Source Data Extended Data Fig. 4 Raw data used for Extended Data 4.

Source Data Extended Data Fig. 5 Raw data used for Extended Data 5.

Source Data Extended Data Fig. 6 Raw data used for Extended Data 6.

Source Data Extended Data Fig. 7 Raw data used for Extended Data 7.

Source Data Extended Data Fig. 8 Raw data used for Extended Data 8.

## Data Availability

Source data for Figs. 1–8 and Extended Data Figs. 1–10 are provided with the paper. The ISB 2001 sequence is pending a patent submission publication. The crystal structures of CD38 in complex with the Fab fragments of daratumumab and isatuximab are available in the PDB under accession codes 7DHA and 4CMH, respectively. All other information is available from the corresponding author on reasonable request. Requests will be processed within 30 days. Source data are provided with this paper.
