# Supplementary material for: ISB 2001 trispecific T cell engager shows strong tumor cytotoxicity and overcomes immune escape mechanisms of multiple myeloma cells
Source: Nat Cancer. 2024 Sep 11;5(10):1494–514. doi: 10.1038/s43018-024-00821-1 (PMC11505469; doi:10.1038/s43018-024-00821-1)
Supplement: Supplementary file 1 — Supplementary Figs. 1 and 2. [file 43018_2024_821_MOESM1_ESM.pdf]

nature cancer

Supplementary information https://doi.org/10.1038/s43018-024-00821-1

ISB 2001 trispecific T cell engager shows strong tumor cytotoxicity and overcomes immune escape mechanisms of multiple myeloma cells

In the format provided by the authors and unedited

Supplementary Figures

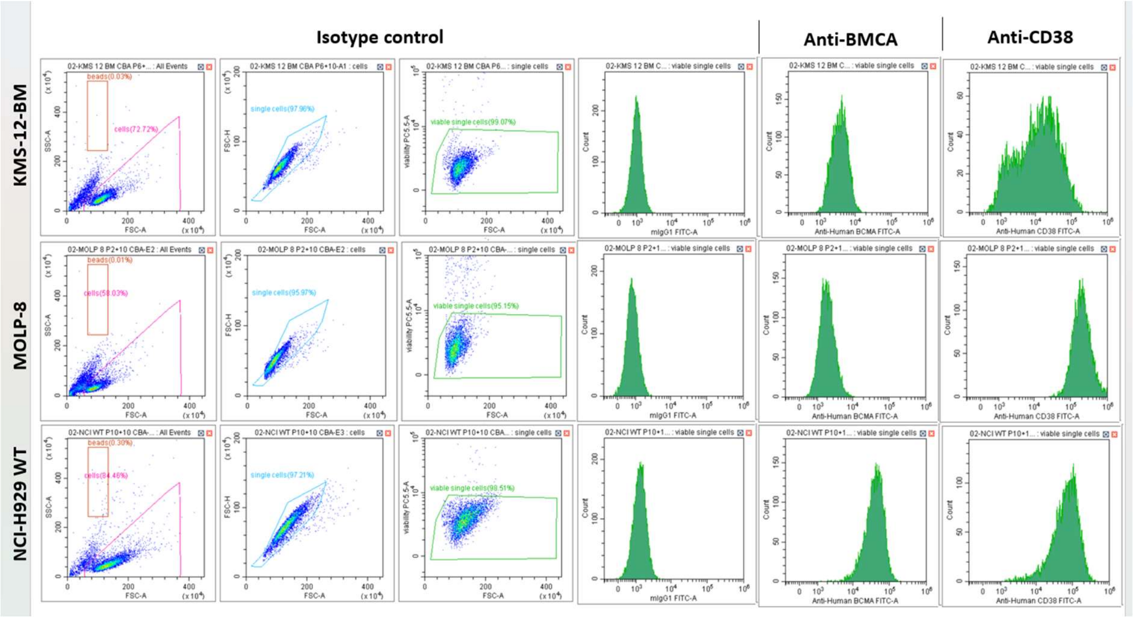

Supplementary Figure 1 (support Raw data of ED 3F). Gating strategy for assessment of the BCMA and CD38 human antibody binding capacity (BioCytex) on three MM cell lines (KMS-12-BM, NCI-H929 and MOLP-8) .

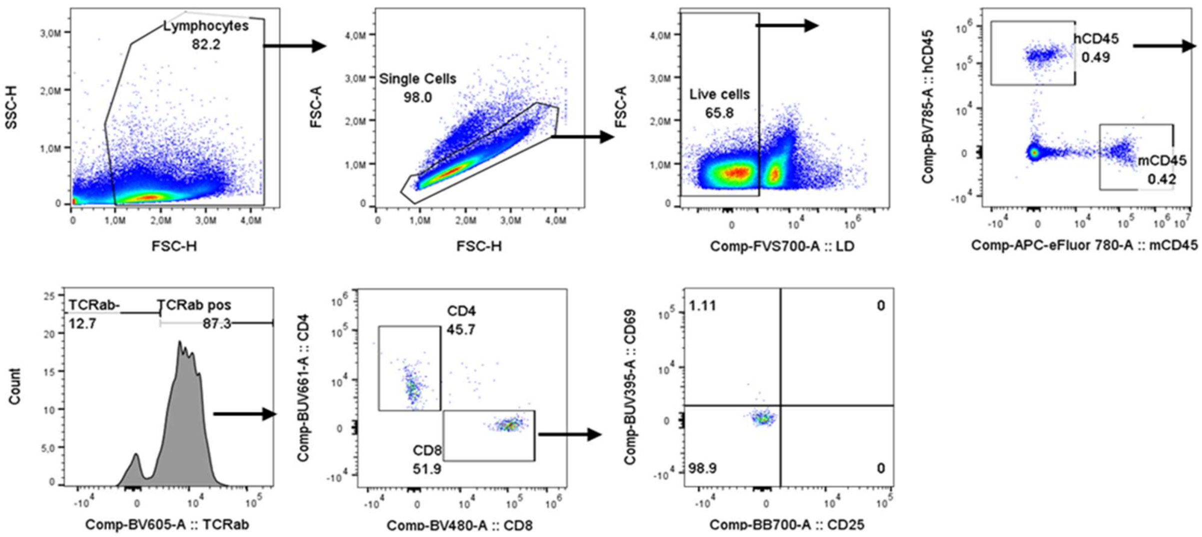

Supplementary Figure 2 (Support Raw data of ED 7B). Full Flow Cytometry gating strategy to identify % CD25+ or % CD69 of CD8+ T cells in Spleen at day 2.
