# Supplementary material for: ISB 2001 trispecific T cell engager shows strong tumor cytotoxicity and overcomes immune escape mechanisms of multiple myeloma cells
Source: Nat Cancer. 2024 Sep 11;5(10):1494–514. doi: 10.1038/s43018-024-00821-1 (PMC11505469; doi:10.1038/s43018-024-00821-1)
Supplement: Supplementary file 2 — Reporting Summary [file 43018_2024_821_MOESM2_ESM.pdf]

nature portfolio

Corresponding author(s): Mario Perro

Last updated by author(s): Jul 22, 2024

Reporting Summary

Nature Portfolio wishes to improve the reproducibility of the work that we publish. This form provides structure for consistency and transparency in reporting. For further information on Nature Portfolio policies, see our Editorial Policies and the Editorial Policy Checklist.

Please do not complete any field with "not applicable" or n/a. Refer to the help text for what text to use if an item is not relevant to your study.
For final submission: please carefully check your responses for accuracy; you will not be able to make changes later.

Statistics

For all statistical analyses, confirm that the following items are present in the figure legend, table legend, main text, or Methods section.

n/a	Confirmed	
☐	☒	The exact sample size (n) for each experimental group/condition, given as a discrete number and unit of measurement
☐	☒	A statement on whether measurements were taken from distinct samples or whether the same sample was measured repeatedly
☐	☒	The statistical test(s) used AND whether they are one- or two-sided<br>*Only common tests should be described solely by name; describe more complex techniques in the Methods section.*
☒	☐	A description of all covariates tested
☐	☒	A description of any assumptions or corrections, such as tests of normality and adjustment for multiple comparisons
☐	☒	A full description of the statistical parameters including central tendency (e.g. means) or other basic estimates (e.g. regression coefficient) AND variation (e.g. standard deviation) or associated estimates of uncertainty (e.g. confidence intervals)
☐	☒	For null hypothesis testing, the test statistic (e.g. F, t, r) with confidence intervals, effect sizes, degrees of freedom and P value noted<br>*Give P values as exact values whenever suitable.*
☒	☐	For Bayesian analysis, information on the choice of priors and Markov chain Monte Carlo settings
☒	☐	For hierarchical and complex designs, identification of the appropriate level for tests and full reporting of outcomes
☒	☐	Estimates of effect sizes (e.g. Cohen's d, Pearson's r), indicating how they were calculated

Our web collection on statistics for biologists contains articles on many of the points above.

Software and code

Policy information about availability of computer code

Data collection

INSTRUMENTS
Biacore 8K+ instrument (Cytiva)
Biacore T200 instrument (Cytiva)
Octet Red96E instrument (Sartorius)
HPLC Alliance 2695 (Waters)
MicroCal PEAQ-DSC differential scanning calorimeter (Malvern Instruments)
Beckman Coulter PA 800 system with DAD/PDA detector (Diode Array Detector/Photodiode Array Detector PDA)
Cell counter Logos Biosystems LUNA-FL™ Automated Fluorescence Cell Counter
Flow Cytometer Beckman Coulter Cytoflex S or LX
Flow Cytometer Sartorius iQue Screener Plus
LSRFortessa
Incucyte S3 with 20X objective
Zeiss LSM800 inverted confocal microscope
Microplate Reader Synergy™ NEO HTS
SOFTWARES
Biacore Insight control software v3.0 (Cytiva)
Biacore T200 control sofware v3.2 (Cytiva)
Octet Data Acquisition software 11.1 (Sartorius)
Empower 3 sofware FR4 (Waters)
MicroCal PEAQ-DSC software v1.61 (Malvern)
SpectroFlow v3.3.0

Beckman Coulter PA 800 system software (32 Karat software)

Data analysis

GraphPad Prism v. 9.0
JMP v15
FlowJo, v10.8.1
CytExpert 2.5.0.77
iQue Forecyt v9
FACSDiva
Imaris version9
LEGENDplex Online tool
Microsoft Excel
Biacore Insight Evaluation software v3.0 (Cytiva)
Biacore T200 Evaluation sofware v3.2 (Cytiva)
Octet Data Analysis HT 11.1 (Sartorius)
Empower 3 sofware FR4 (Waters) (SE-HPLC + cGE)
MicroCal PEAQ-DSC software v1.61
Phoenix® WinNonlin version 8.3 (Certara, USA)
PyMol v2.5 (Schrödinger, LLC)

For manuscripts utilizing custom algorithms or software that are central to the research but not yet described in published literature, software must be made available to editors and reviewers. We strongly encourage code deposition in a community repository (e.g. GitHub). See the Nature Portfolio guidelines for submitting code & software for further information.

Data

Policy information about availability of data

All manuscripts must include a data availability statement. This statement should provide the following information, where applicable:

- Accession codes, unique identifiers, or web links for publicly available datasets
- A description of any restrictions on data availability
- For clinical datasets or third party data, please ensure that the statement adheres to our policy

All Source data for Figure 1-8 and Extended Data 1-10 are provided with the paper. ISB 2001 sequence is pending patent submission publication. Crystal structures of CD38 in complex with the Fab fragments of daratumumab and isatuximab are available in the protein data bank (PDB) (https://doi.org/10.2210/pdb7DHA/pdb and https://doi.org/10.2210/pdb4CMH/pdb, respectively). All other information are available from the corresponding author on reasonable request. Requests will be processed within 30 days.

Research involving human participants, their data, or biological material

Policy information about studies with human participants or human data. See also policy information about sex, gender (identity/presentation), and sexual orientation and race, ethnicity and racism.

Reporting on sex and gender	We received human samples from healthy donors and patients in an anonymized setting. So no information about sex are available. In addition, due to limited availability of fresh primary patients' material, samples were used as they were available and priority was given to different treatment history rather the the gender of each patients.
Reporting on race, ethnicity, or other socially relevant groupings	The findings in this study were not involved in race, ethnicity, or other socially relevant grouping.
Population characteristics	No data on population characteristics was collected nor used.
Recruitment	Healthy donors were recruited by Transfusion Interregionale CRS (Lausanne, Switzerland). Bone marrow aspirates (BMA) or peripheral blood samples from MM patients were obtained from University Hospital Geneva (Geneva ethical committee: 2021-02416), Oxford University Hospitals (Oxford Clinical Research Ethics Committee (17/SC/0572) and the HaemBiobank Governance Committee (BBProj-27.0 and BBProj-13.0)) and Nantes Université (MYRACLE cohort NCT03807128) with informed consent under each site ethical approvals.
Ethics oversight	All research on healthy human donor was approved by transfusion Interregionale CRS, with all donors provided written informed consent in accordance with the Declaration of Helsinki and the protocol of the local institutional review board, the Medical Ethics Committee of Transfusion Interregionale CRS.<br>All research on multiple myeloma patient's samples were performed in accordance with ethical approvals with collaborating institutes. Oxford Clinical Research: The study was approved by the Oxford Clinical Research Ethics Committee (17/SC/0572) and the HaemBiobank Governance Committee (BBProj-27.0 and BBProj-13.0). Nantes: all samples were obtained from the cohort MYRACLE (Benanina L et al., BMC Cancer, 2019), NCT03807128. University Hospital Geneve (HUG): ethical approval number 2021-02416. Written informed consent was obtained from all patients.

Note that full information on the approval of the study protocol must also be provided in the manuscript.

Field-specific reporting

Please select the one below that is the best fit for your research. If you are not sure, read the appropriate sections before making your selection.

☒ Life sciences ☐ Behavioural & social sciences ☐ Ecological, evolutionary & environmental sciences

For a reference copy of the document with all sections, see nature.com/documents/nr-reporting-summary-flat.pdf

Life sciences study design

All studies must disclose on these points even when the disclosure is negative.

Sample size	For in vitro experiments, no statistical methods were used to pre-determine sample.Instead sample size was determined empirically according to previous knowledge of the variation in experimental setup.<br>For in vivo studies, 7-9 mice/group were used to detect significancy between groups, based on power calculations using G*Power (90 % power and 0.05 error prob).
Data exclusions	EC50 values were excluded from analysis when the R2 of the non-linear regression fitting curve was below 0.7, when the observed maximum response was below 25% or when the calculated EC50 values were out of the range of the tested concentrations. For binding measurements by flow cytometry, values were excluded for KD calculations when a hook effect was observed.
Replication	Number of biological replicates, independent experiments performed as well as statistical analysis performed are stated in all figure legends. Overall, all experiments were performed using replications as needed to obtain a valid answer to the scientific questions.
Randomization	For in vivo tumor models, mice randomization was performed when tumors reached an average of 150 mm3 based on the tumor volume.
Blinding	Data collection and analysis were performed blind for the outsourced in vivo experiments (NCI-H929 model) performed in Crown Bioscience Inc (Beijing, China) and The Jackson laboratories (Bar Harbor, Maine, USA) for tumor control and PK experiments. Data collection and analysis was not blinded for the other models/experiments.

Reporting for specific materials, systems and methods

We require information from authors about some types of materials, experimental systems and methods used in many studies. Here, indicate whether each material, system or method listed is relevant to your study. If you are not sure if a list item applies to your research, read the appropriate section before selecting a response.

Materials & experimental systems

n/a	Involved in the study
☐	☒ Antibodies
☐	☒ Eukaryotic cell lines
☒	☐ Palaeontology and archaeology
☐	☒ Animals and other organisms
☒	☐ Clinical data
☒	☐ Dual use research of concern
☒	☐ Plants

Methods

n/a	Involved in the study
☒	☐ ChIP-seq
☐	☒ Flow cytometry
☒	☐ MRI-based neuroimaging

Antibodies

Antibodies used

All the commercially available antibodies used in this study (clone, catalog number, supplier, dilution or concentration used and experiment where they have been used) can be found in Methods Table 2 in Supplementary Information file.

Validation

All antibodies were titrated with a dose-response from at least 1/25 with a serial dilution of 1/2 up to 1/3200 on positive cells and non-expressing cells. Choice of the optimal antibody dilution is based on the stain index calculation Stain Index (SI)=(MFI of positive population -MFI of negative population)/(2*SD of negative population)

Eukaryotic cell lines

Policy information about cell lines and Sex and Gender in Research

Cell line source(s)

For in vitro/in vivo experiments, KMS-12-BM (ACC551) and MOLP-8 (ACC569) were purchased from DSMZ. NCI-H929 (9505041) was purchased from Sigma-Aldrich. NCI-H929 deficient for BCMA or CD38 were generated in-house using CRISPR/Cas9 technology (See Methods section for details). For molecules production, CHO-S cells (cGMP banked) were purchased from Invitrogen and HD-BIOP3 from Horizon Discovery.

Authentication

Authentication was performed using short tandem repeat (STR) analysis evaluated by by Microsynth (Balgach, Switzerland) at passage 5 and passage 15 according to Microsynth guidelines

Mycoplasma contamination

All cell lines were tested negative for mycoplasma

Commonly misidentified lines
(See ICLAC register)

No commonly misidentified cell lines were used (according to ICLAC register version 10)

Animals and other research organisms

Policy information about studies involving animals; ARRIVE guidelines recommended for reporting animal research, and Sex and Gender in Research

Laboratory animals	Mouse (Mus musculus)<br>Tumor growth experiments were performed in the following mouse strains: 6-7-week-old NSG (NOD.Cg-Prkdcscid Il2rgtm1Wjl/SzJ), 24-30-weeks old HIS-NXG (human immunized system- NOD-Prkdcscid-IL2rgTm1/Rj, reconstituted with human cord blood CD34+ cells) and 6-7 week-old NCG (NOD-Prkdcem26Cd52Il2rgem26Cd22/NjuCrl).<br>PK studies were performed in the following mouse strains: 8-9-week-old NCG (NOD-Prkdcem26Cd52Il2rgem26Cd22/NjuCrl) and hFcRn Tg32 SCID mice (B6.Cg-Fcgrttm1Dcr Prkdcscid Tg(FCGRT)32Dcr/DcrJ; JAX stock# 018441).<br>All mice were maintained under standardized environmental conditions in rodent cages (20-26°C temperature, 40-70% relative humidity, 12 hours light dark cycle). Mice received irradiated food and bedding and 0.22 µm-filtered drinking water ad libitum.
Wild animals	No wild animals involved
Reporting on sex	Due to the need for social housing AND randomization of mice based on tumor size female mice were used for studies with tumors to respect the 3Rs and minimize animal stress while minimizing the risk of experimenter error if mice with different treatments were co-housed.<br>For PK experiments mice of either sex were used based on availability and bodyweight (to ensure ethical blood sampling as maximum blood volume is determined by weight).
Field-collected samples	no field samples
Ethics oversight	All work conducted at Ichnos sciences was reviewed by the Vaud cantonal committee for animal experimentation and the Swiss federal authorities. Work conducted at Jackson laboratories was overseen by their IACUC and work conducted at Crown biosciences was overseen by their IACUC. Both Crown and Jackson labs are AAALAC accredited organizations.

Note that full information on the approval of the study protocol must also be provided in the manuscript.

Flow Cytometry

Plots

Confirm that:

☒ The axis labels state the marker and fluorochrome used (e.g. CD4-FITC).

☒ The axis scales are clearly visible. Include numbers along axes only for bottom left plot of group (a 'group' is an analysis of identical markers).

☒ All plots are contour plots with outliers or pseudocolor plots.

☒ A numerical value for number of cells or percentage (with statistics) is provided.

Methodology

Sample preparation	Sample preparation for flow cytometry is described in details in the M&M section of the manuscript in the flow cytometry assays section. For quantification of surface antibodies bound to cells (ABC), expression levels of CD38 and BCMA were determined using Qifikit® (for mouse primary antibodies) or Biocytex® kit (for human primary antibodies) according to the manufacturer's instructions. The number of primary antibodies bound on the cells (sABC value) was determined by interpolation using a calibration curve. For in vitro and in vivo experiments, at the readout time point, cells were resuspended either in PBS, washed and labelled with Live/Dead dyes, or resuspended in FACS buffer (PBS containing 2.5% FCS 2mM EDTA), washed and stained with an antibody mix diluted in FACS buffer. After 20-30 minutes of staining at 4°C in the dark, cell suspensions were washed with FACS buffer twice and resuspended in FACS buffer or Sytox dyes or DAPI and the readout was performed using Flow Cytometer (Cytoflex-S or -LX (BC), iQueScreenerPlus (Sartorius) or Aurora (Cytek)).
Instrument	Cytoflex S, Beckman Coulter<br>Flow Cytometer Sartorius iQue Screener Plus<br>Aurora (Cytek)
Software	FlowJo, v10.8.1<br>CytExpert 2.5.0.77<br>iQue Forecyt
Cell population abundance	T cells purities after isolation at the start of assay > 95%.
Gating strategy	In general, FSC/SSC plot was used to gate cells and exclude debris; FSC-A/FSC-H to gate single cells; Live cells: FSC-A vs Live Dead; CD138 vs CD38 on single live cells to identify plasma cells .Gating strategies are provided in the source data.

☒ Tick this box to confirm that a figure exemplifying the gating strategy is provided in the Supplementary Information.

This checklist template is licensed under a Creative Commons Attribution 4.0 International License, which permits use, sharing, adaptation, distribution and reproduction in any medium or format, as long as you give appropriate credit to the original author(s) and the source, provide a link to the Creative Commons license, and indicate if changes were made. The images or other third party material in this article are included in the article's Creative Commons license, unless indicated otherwise in a credit line to the material. If material is not included in the article's Creative Commons license and your intended use is not permitted by statutory regulation or exceeds the permitted use, you will need to obtain permission directly from the copyright holder. To view a copy of this license, visit http://creativecommons.org/licenses/by/4.0/
